# Supplementary material for: The DNA-PAINT palette: a comprehensive performance analysis of fluorescent dyes
Source: Nat Methods. 2024 Aug 7;21(9):1755–62. doi: 10.1038/s41592-024-02374-8 (PMC11399092; doi:10.1038/s41592-024-02374-8)
Supplement: Supplementary file 1 — Supplementary Figs. 1–18 and Tables 1–5. [file 41592_2024_2374_MOESM1_ESM.pdf]

nature methods

Supplementary information https://doi.org/10.1038/s41592-024-02374-8

The DNA-PAINT palette: a comprehensive performance analysis of fluorescent dyes

In the format provided by the authors and unedited

Supplementary Information

The DNA-PAINT palette: A comprehensive performance analysis of fluorescent dyes

Philipp R. Steen[1,2], Eduard M. Unterauer[1,2], Luciano A. Masullo[2], Jisoo Kwon[2], Ana Perovic[2], Kristina Jevdokimenko[3], Felipe Opazo[3,4,5], Eugenio F. Fornasiero[3,6], and Ralf Jungmann[1,2]

[1]Faculty of Physics and Center for Nanoscience, Ludwig Maximilian University, Munich, Germany. [2]Max Planck Institute of Biochemistry, Martinsried, Germany. [3]Institute of Neuro- and Sensory Physiology, University Medical Center Göttingen, Göttingen, Germany. [4]Center for Biostructural Imaging of Neurodegeneration (BIN), University Medical Center Göttingen, Göttingen, Germany. [5]NanoTag Biotechnologies GmbH, Göttingen, Germany. [6]Department of Life Sciences, University of Trieste, Trieste, Italy. Correspondence should be addressed to R.J. (jungmann@biochem.mpg.de).

**Supplementary Figure 1**	Dye analysis, Alexa Fluor 488
**Supplementary Figure 2**	Dye analysis, Abberior Star 488
**Supplementary Figure 3**	Dye analysis, Atto488
**Supplementary Figure 4**	Dye analysis, CF488A
**Supplementary Figure 5**	Dye analysis, Atto565
**Supplementary Figure 6**	Dye analysis, CF568
**Supplementary Figure 7**	Dye analysis, Cy3B
**Supplementary Figure 8**	Dye analysis, Janelia Fluor 585
**Supplementary Figure 9**	Dye analysis, Alexa Fluor 647
**Supplementary Figure 10**	Dye analysis, Abberior Star 635p
**Supplementary Figure 11**	Dye analysis, Atto643
**Supplementary Figure 12**	Dye analysis, Atto647N
**Supplementary Figure 13**	Dye analysis, Atto655
**Supplementary Figure 14**	Dye analysis, CF640R
**Supplementary Figure 15**	Dye analysis, CF660R
**Supplementary Figure 16**	Dye analysis, Cy5
**Supplementary Figure 17**	Dye analysis, Cy5B
**Supplementary Figure 18**	Dye analysis, Janelia Fluor 646
**Supplementary Table 1**	Imaging Parameters
**Supplementary Table 2**	Sequences
**Supplementary Table 3**	Antibodies
**Supplementary Table 4**	Cross-talk in 3-channel imaging
**Supplementary Table 5**	DNA origami scaffold sequence

Supplementary Figs. 1-18 | Dye analysis. a, Absorption (grey) and emission (black) spectra with QY where available. **b,** Achievable precision illustrated by 20 nm DNA origami. **c,** Emission photon distribution and mean number of photons ($\overline{N}$) per 100 ms. **d,** Bright time distribution and mean bright time ($\overline{\tau_b}$). **e,** Dye stability expressed as localizations over time (per 20 seconds per docking site) over the course of the measurement. **f,** Mean DNA origami-based dye analysis results for all tested buffer conditions. Spectra, quantum yields (QY) where available and extinction coefficients from respective manufacturers. Uncertainties represent s.d. from 3 repeat measurements. **g,** Representative NPC (Nup96 labeled) illustrating achievable resolution in 3D, in cells. Left: top view, right: side view. **h,** Emission photon distribution. **i,** Comparison of representative nuclear (left) vs. cytoplasmic (right) localizations. **j,** Mean dye analysis results for cellular experiments. Uncertainties represent s.d. from 3 repeat measurements.

DNA origami imaged using Alexa Fluor 488 with Trolox

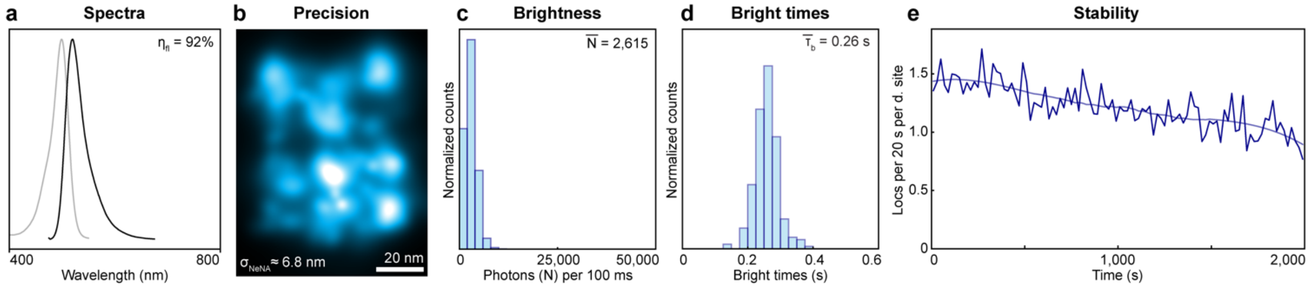

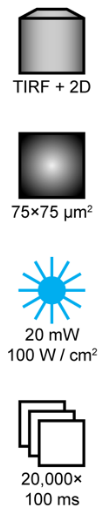

f DNA origami average results for all buffer conditions

Dye	Ex. max. (nm)	Em. max. (nm)	QY	Ext. (×10³ M⁻¹ cm⁻¹)	Buffer additive	Precision (nm)	Photons per 100 ms	Bright time (s)	Signal to background ratio	Localization drop (%)	Destroyed binding sites (%)
Alexa Fluor 488	499	520	0.92	73	-	8.7 ± 1.5	1908 ± 209	0.21 ± 0.01	2.3 ± 0.2	28.3 ± 11.0	3.7 ± 1.6
					Trolox	6.3 ± 0.5	2811 ± 290	0.24 ± 0.03	2.7 ± 0.5	33.6 ± 3.5	7.4 ± 1.8
					PCA PCD Trolox	8.1 ± 2.3	1979 ± 1131	0.23 ± 0.01	1.9 ± 0.2	24.5 ± 16.0	3.5 ± 1.3

U2OS Nup96 imaged using Alexa Fluor 488 with Trolox

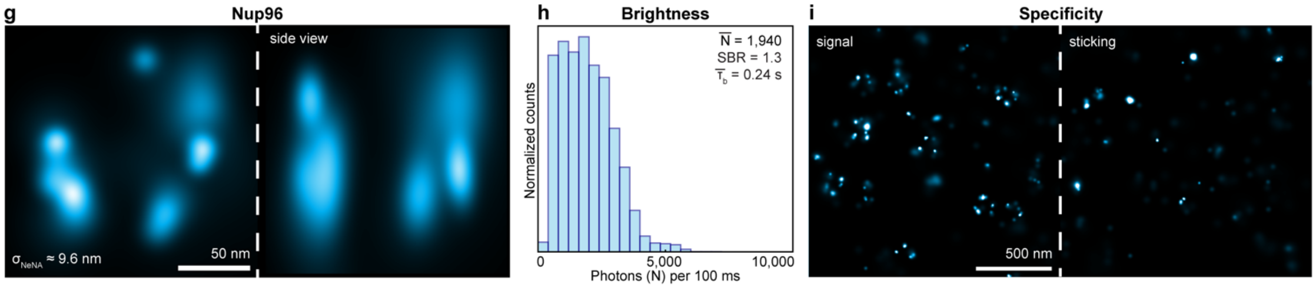

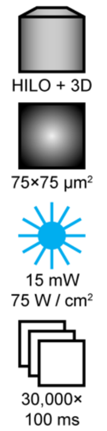

j U2OS Nup96 average results with Trolox

Dye	Buffer additive	Precision (nm)	Photons per 100 ms	Bright time (s)	Signal to background ratio	Localization drop (%)	Relative specificity
Alexa Fluor 488	Trolox	9.3 ± 0.4	2191 ± 860	0.23 ± 0.01	1.3 ± 0.3	21.2 ± 7.0	0.42 ± 0.14

Supplementary Figure 1 | Dye analysis, Alexa Fluor 488

DNA origami imaged using Abberior Star 488 with Trolox

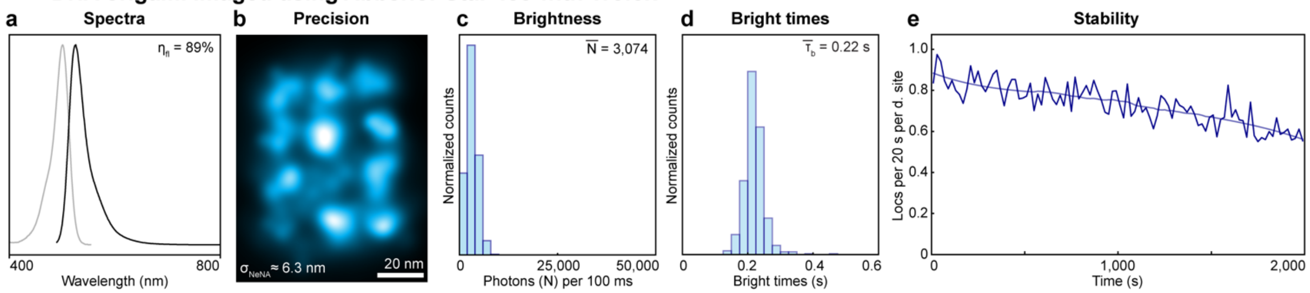

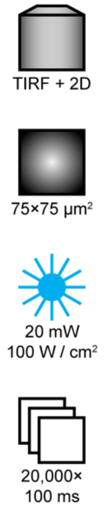

f DNA origami average results for all buffer conditions

Dye	Ex. max. (nm)	Em. max. (nm)	QY	Ext. (×10³ M⁻¹ cm⁻¹)	Buffer additive	Precision (nm)	Photons per 100 ms	Bright time (s)	Signal to background ratio	Localization drop (%)	Destroyed binding sites (%)
Abberior Star 488	503	524	0.89	65	-	7.8 ± 0.0	1874 ± 137	0.17 ± 0.00	2.0 ± 0.6	28.2 ± 19.1	8.7 ± 6.4
					Trolox	6.3 ± 0.6	2969 ± 365	0.21 ± 0.02	2.7 ± 0.7	27.6 ± 4.5	9.1 ± 2.7
					PCA PCD Trolox	8.9 ± 2.7	2317 ± 910	0.22 ± 0.00	1.9 ± 0.2	8.3 CI (0.0, 20.0)	3.2 ± 0.0

U2OS Nup96 imaged using Abberior Star 488 with Trolox

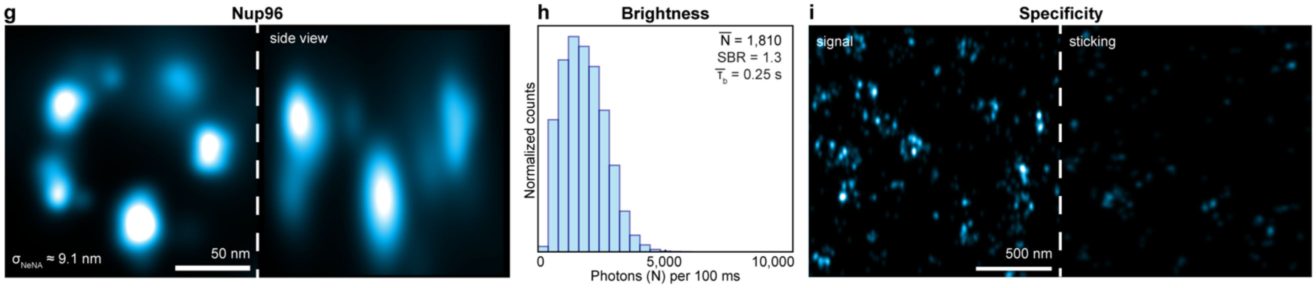

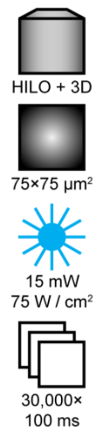

j U2OS Nup96 average results with Trolox

Dye	Buffer additive	Precision (nm)	Photons per 100 ms	Bright time (s)	Signal to background ratio	Localization drop (%)	Relative specificity
Abberior Star 488	Trolox	11.5 ± 2.9	1532 ± 274	0.23 ± 0.03	1.0 ± 0.2	11.4 ± 10.5	0.55 ± 0.11

Supplementary Figure 2 | Dye analysis, Abberior Star 488

DNA origami imaged using Atto488 with Trolox

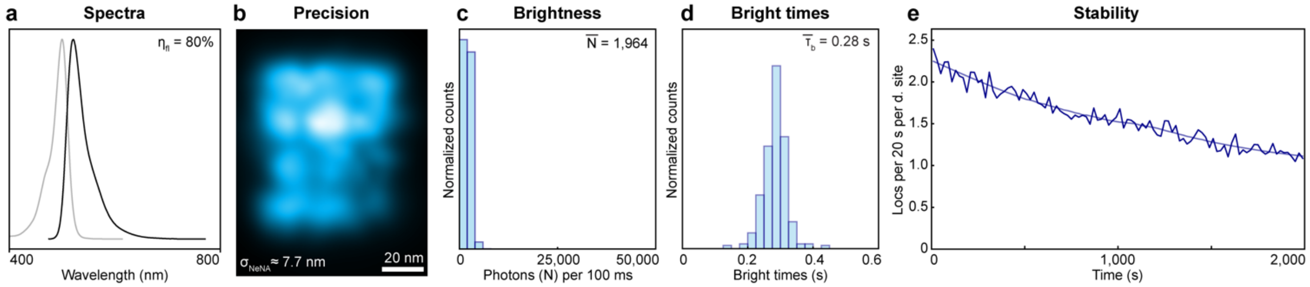

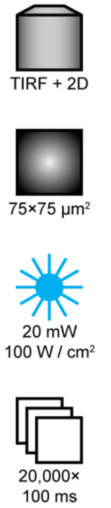

f **DNA origami average results for all buffer conditions**

Dye	Ex. max. (nm)	Em. max. (nm)	QY	Ext. (×10³ M⁻¹ cm⁻¹)	Buffer additive	Precision (nm)	Photons per 100 ms	Bright time (s)	Signal to background ratio	Localization drop (%)	Destroyed binding sites (%)
Atto488	500	520	0.8	90	-	8.7 ± 0.8	1849 ± 465	0.22 ± 0.01	1.4 ± 0.1	48.3 ± 8.6	19.3 ± 0.6
					Trolox	7.4 ± 0.3	2073 ± 249	0.27 ± 0.02	1.6 ± 0.2	44.3 ± 2.8	17.1 ± 3.6
					PCA PCD Trolox	8.4 ± 0.2	1804 ± 264	0.27 ± 0.00	1.4 ± 0.2	16.4 ± 8.3	9.2 ± 0.3

U2OS Nup96 imaged using Atto488 with Trolox

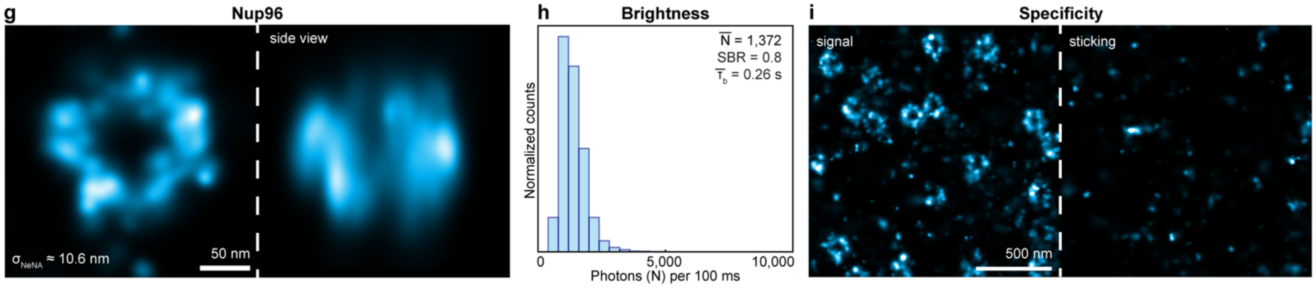

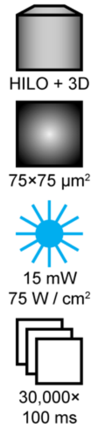

j **U2OS Nup96 average results with Trolox**

Dye	Buffer additive	Precision (nm)	Photons per 100 ms	Bright time (s)	Signal to background ratio	Localization drop (%)	Relative specificity
Atto488	Trolox	11.4 ± 1.4	1388 ± 181	0.26 ± 0.00	0.8 ± 0.1	16.4 ± 3.5	0.71 ± 0.19

Supplementary Figure 3 | Dye analysis, Atto488

DNA origami imaged using CF488A with Trolox

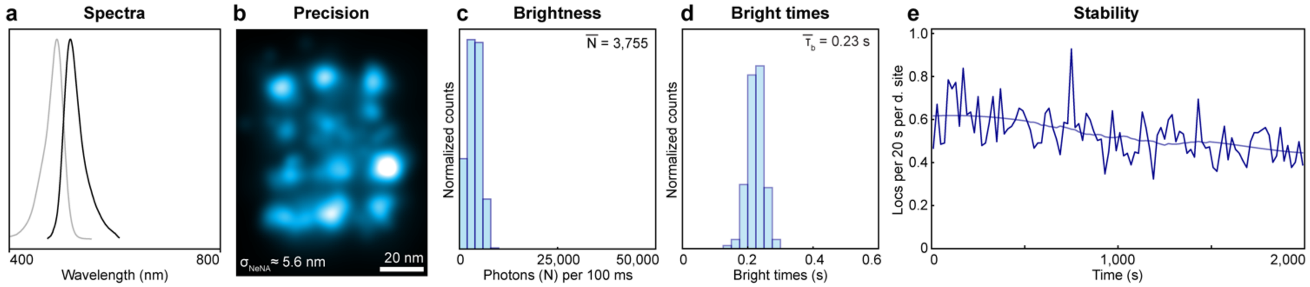

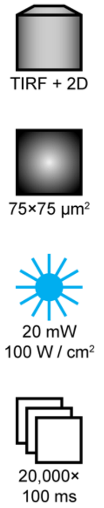

f DNA origami average results for all buffer conditions

Dye	Ex. max. (nm)	Em. max. (nm)	QY	Ext. (×10³ M⁻¹ cm⁻¹)	Buffer additive	Precision (nm)	Photons per 100 ms	Bright time (s)	Signal to background ratio	Localization drop (%)	Destroyed binding sites (%)
CF488A	490	515	-	70	-	6.9 ± 0.8	2492 ± 578	0.16 ± 0.00	2.0 ± 0.2	32.0 ± 0.4	8.9 ± 2.8
					Trolox	5.4 ± 0.6	3879 ± 732	0.20 ± 0.03	3.2 ± 0.8	33.7 ± 10.4	8.3 ± 3.6
					PCA PCD Trolox	5.9 ± 0.8	3901 ± 412	0.22 ± 0.00	2.5 ± 0.0	11.2 ± 5.9	5.4 ± 2.1

U2OS Nup96 imaged using CF488A with Trolox

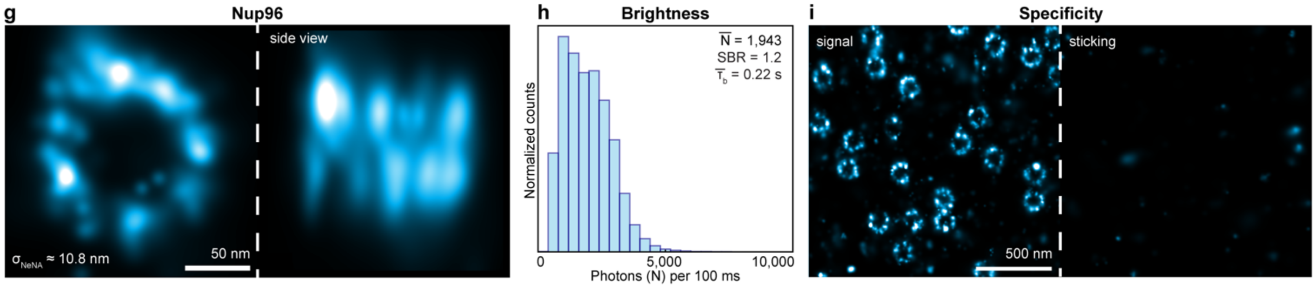

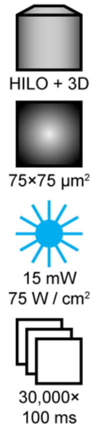

j U2OS Nup96 average results with Trolox

Dye	Buffer additive	Precision (nm)	Photons per 100 ms	Bright time (s)	Signal to background ratio	Localization drop (%)	Relative specificity
CF488A	Trolox	10.7 ± 0.1	2240 ± 305	0.21 ± 0.01	1.3 ± 0.1	15.5 ± 7.4	0.48 ± 0.07

Supplementary Figure 4 | Dye analysis, CF488A

DNA origami imaged using Atto565 with Trolox

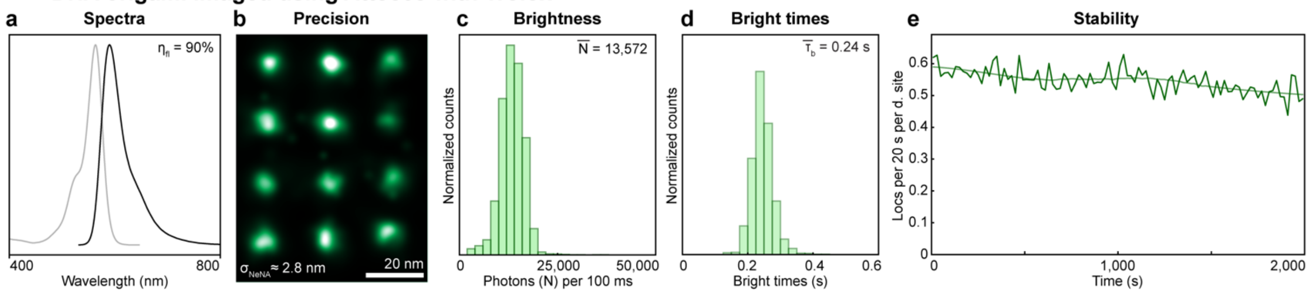

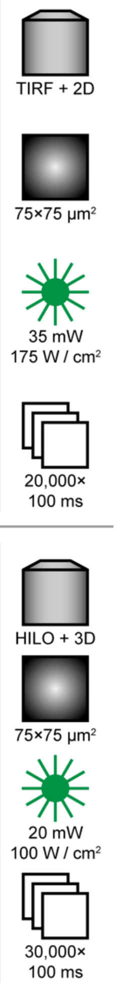

f DNA origami average results for all buffer conditions

Dye	Ex. max. (nm)	Em. max. (nm)	QY	Ext. (×10³ M⁻¹ cm⁻¹)	Buffer additive	Precision (nm)	Photons per 100 ms	Bright time (s)	Signal to background ratio	Localization drop (%)	Destroyed binding sites (%)
Atto565	564	590	0.9	120	-	3.2 ± 0.2	9804 ± 1907	0.24 ± 0.01	10.3 ± 0.3	18.8 ± 4.0	3.6 ± 1.6
					Trolox	2.9 ± 0.1	11600 ± 1896	0.24 ± 0.01	11.1 ± 2.8	16.4 ± 4.0	1.3 ± 0.5
					PCA PCD Trolox	3.3 ± 0.2	10295 ± 3494	0.23 ± 0.01	11.3 ± 0.3	12.1 ± 0.7	5.8 ± 4.5

U2OS Nup96 imaged using Atto565 with Trolox

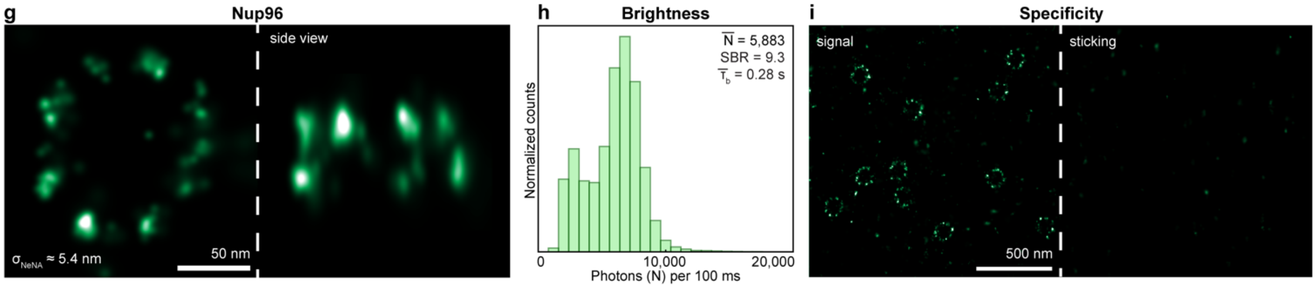

j U2OS Nup96 average results with Trolox

Dye	Buffer additive	Precision (nm)	Photons per 100 ms	Bright time (s)	Signal to background ratio	Localization drop (%)	Relative specificity
Atto565	Trolox	6.0 ± 0.6	6287 ± 917	0.28 ± 0.01	8.4 ± 0.9	4.2 CI (0.0, 9.3)	0.87 ± 0.39

Supplementary Figure 5 | Dye analysis, Atto565

DNA origami imaged using CF568

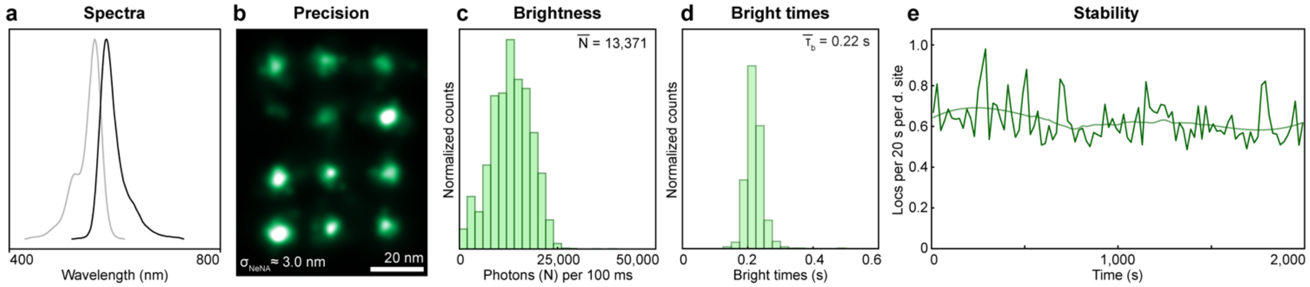

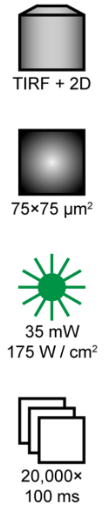

f DNA origami average results for all buffer conditions

Dye	Ex. max. (nm)	Em. max. (nm)	QY	Ext. (×10³ M⁻¹ cm⁻¹)	Buffer additive	Precision (nm)	Photons per 100 ms	Bright time (s)	Signal to background ratio	Localization drop (%)	Destroyed binding sites (%)
CF568	562	583	-	100	-	3.0 ± 0.2	13388 ± 2033	0.21 ± 0.01	10.9 ± 2.5	11.5 ± 1.1	2.3 ± 0.2
					Trolox	3.1 ± 0.2	14729 ± 1465	0.21 ± 0.00	13.6 ± 0.7	6.3 CI (0.0, 10.0)	1.1 CI (0.0, 2.4)
					PCA PCD Trolox	3.8 ± 0.3	10456 ± 264	0.22 ± 0.01	10.8 ± 1.4	6.1 ± 1.8	1.4 ± 0.8

U2OS Nup96 imaged using CF568

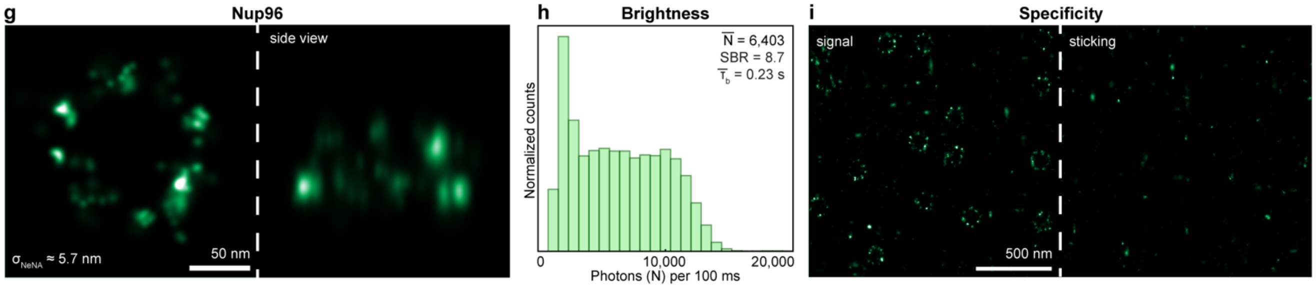

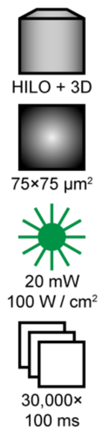

j U2OS Nup96 average results

Dye	Buffer additive	Precision (nm)	Photons per 100 ms	Bright time (s)	Signal to background ratio	Localization drop (%)	Relative specificity
CF568	-	6.0 ± 0.8	6332 ± 62	0.24 ± 0.02	7.1 ± 2.5	2.4 CI (0.0, 6.6)	0.51 ± 0.21

Supplementary Figure 6 | Dye analysis, CF568

DNA origami imaged using Cy3B with PCA / PCD / Trolox

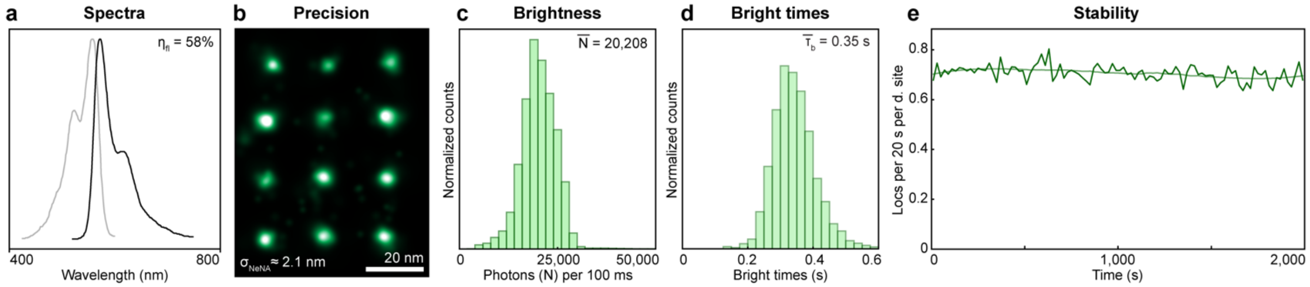

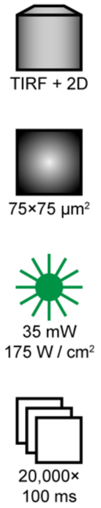

f DNA origami average results for all buffer conditions

Dye	Ex. max. (nm)	Em. max. (nm)	QY	Ext. (×10^3 M^{-1} cm^{-1})	Buffer additive	Precision (nm)	Photons per 100 ms	Bright time (s)	Signal to background ratio	Localization drop (%)	Destroyed binding sites (%)
Cy3B	560	571	0.58	120	-	2.7 ± 0.2	15574 ± 3716	0.30 ± 0.03	14.8 ± 2.5	11.6 ± 7.7	2.1 ± 1.2
					Trolox	2.3 ± 0.2	17653 ± 4508	0.33 ± 0.04	16.0 ± 3.1	4.0 ± 3.9	1.5 ± 0.6
					PCA PCD Trolox	2.0 ± 0.1	23195 ± 3323	0.35 ± 0.01	14.3 ± 2.6	3.7 ± 3.4	2.1 ± 0.3

U2OS Nup96 imaged using Cy3B with PCA / PCD / Trolox

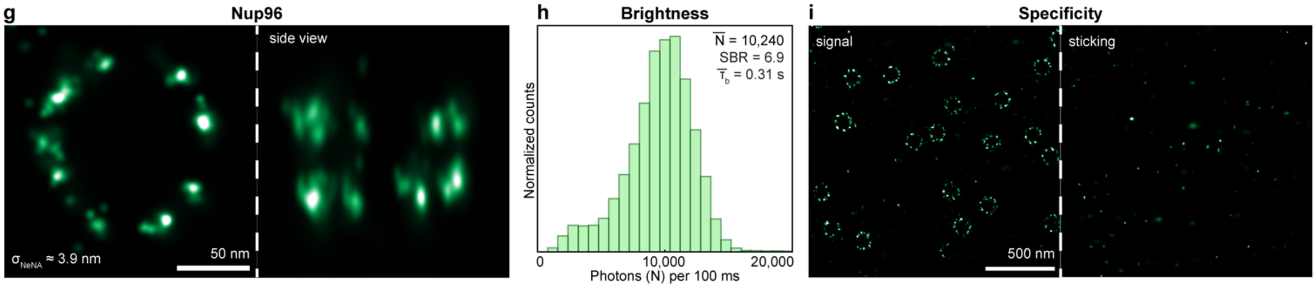

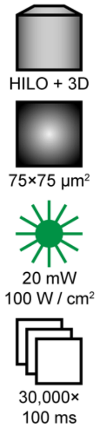

j U2OS Nup96 average results with PCA / PCD / Trolox

Dye	Buffer additive	Precision (nm)	Photons per 100 ms	Bright time (s)	Signal to background ratio	Localization drop (%)	Relative specificity
Cy3B	PCA PCD Trolox	4.5 ± 0.4	6991 ± 2203	0.34 ± 0.03	6.3 ± 1.4	4.9 CI (0.0, 14.3)	1.04 ± 0.09

Supplementary Figure 7 | Dye analysis, Cy3B

DNA origami imaged using Janelia Fluor 585 with Trolox

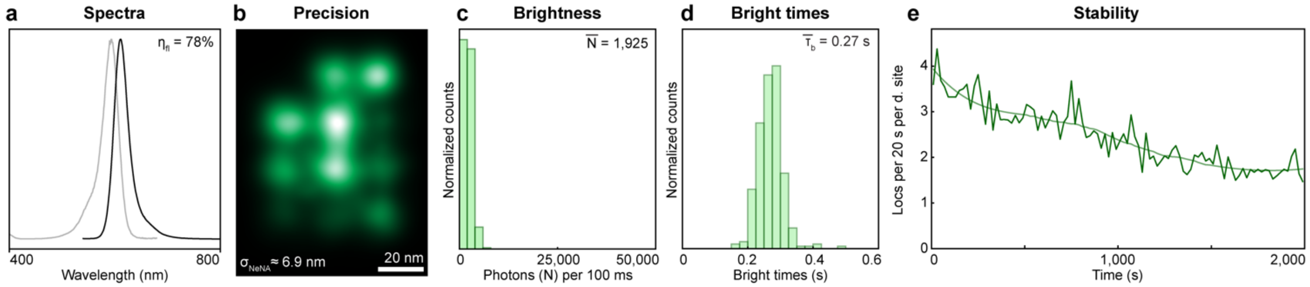

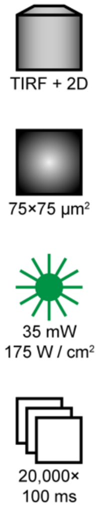

f DNA origami average results for all buffer conditions

Dye	Ex. max. (nm)	Em. max. (nm)	QY	Ext. (×10³ M⁻¹ cm⁻¹)	Buffer additive	Precision (nm)	Photons per 100 ms	Bright time (s)	Signal to background ratio	Localization drop (%)	Destroyed binding sites (%)
					-	7.6 ± 0.6	1791 ± 224	0.28 ± 0.01	3.5 ± 0.1	30.5 CI (0.0, 62.9)	20.2 CI (0.0, 45.3)
Janelia Fluor 585	585	609	0.78	156	Trolox	6.9 ± 1.1	2429 ± 1137	0.26 ± 0.02	3.6 ± 0.3	35.6 ± 28.7	17.7 ± 13.4
					PCA PCD Trolox	8.5 ± 0.6	1715 ± 214	0.26 ± 0.00	3.3 ± 0.0	10.2 CI (0.0, 24.5)	5.8 ± 4.5

U2OS Nup96 imaged using Janelia Fluor 585 with Trolox

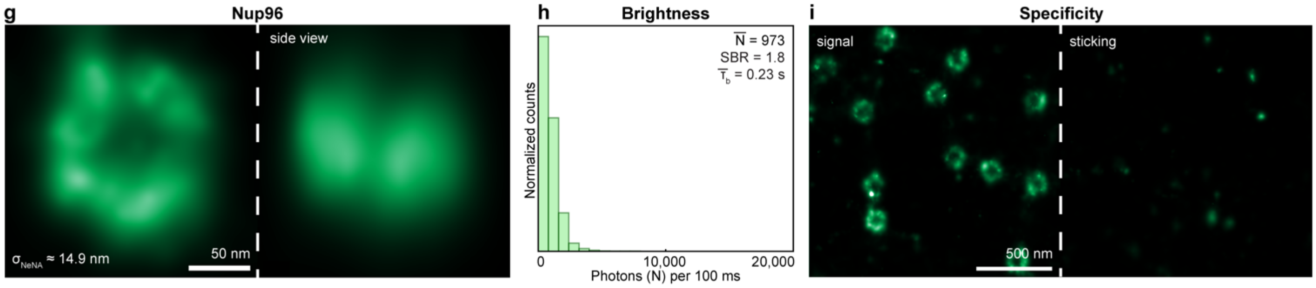

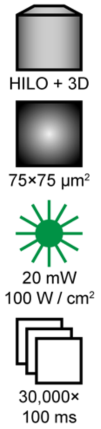

j U2OS Nup96 average results

Dye	Buffer additive	Precision (nm)	Photons per 100 ms	Bright time (s)	Signal to background ratio	Localization drop (%)	Relative specificity
Janelia Fluor 585	Trolox	17.4 ± 2.5	741 ± 219	0.22 ± 0.01	1.0 ± 0.7	8.8 CI (0.0, 19.6)	1.30 ± 0.47

Supplementary Figure 8 | Dye analysis, Janelia Fluor 585

DNA origami imaged using Alexa Fluor 647 with PCA / PCD / Trolox

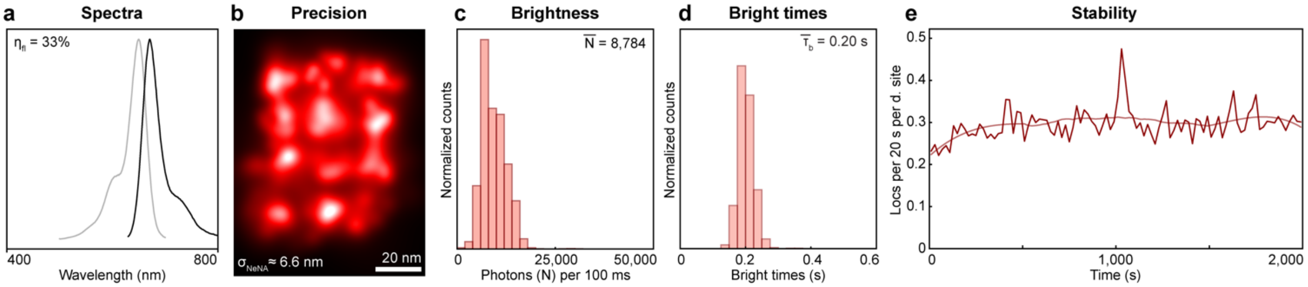

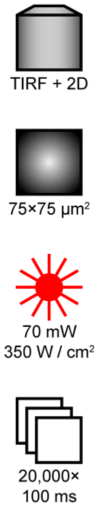

f DNA origami average results for all buffer conditions

Dye	Ex. max. (nm)	Em. max. (nm)	QY	Ext. (×10³ M⁻¹ cm⁻¹)	Buffer additive	Precision (nm)	Photons per 100 ms	Bright time (s)	Signal to background ratio	Localization drop (%)	Destroyed binding sites (%)
Alexa Fluor 647	650	671	0.33	270	-	10.0 ± 1.9	8928 ± 1015	0.18 ± 0.01	1.9 ± 0.3	43.6 ± 20.3	15.6 ± 9.8
					Trolox	9.5 ± 1.2	9293 ± 1622	0.18 ± 0.01	2.3 ± 0.0	41.6 ± 17.3	12.1 ± 8.5
					PCA PCD Trolox	6.3 ± 0.2	10348 ± 1358	0.19 ± 0.01	2.1 ± 0.2	1.4 CI (0.0, 2.9)	1.2 ± 0.1

U2OS Nup96 imaged using Alexa Fluor 647 with PCA / PCD / Trolox

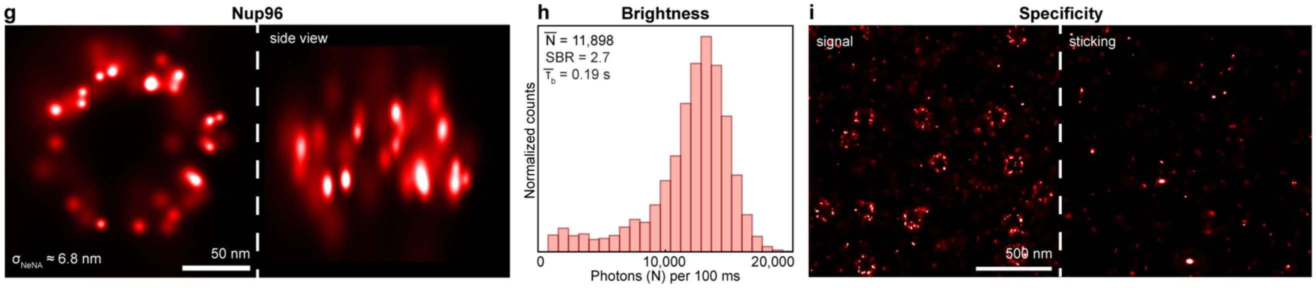

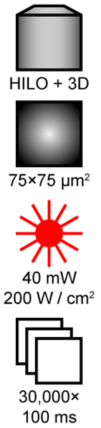

j U2OS Nup96 average results with PCA / PCD / Trolox

Dye	Buffer additive	Precision (nm)	Photons per 100 ms	Bright time (s)	Signal to background ratio	Localization drop (%)	Relative specificity
Alexa Fluor 647	PCA PCD Trolox	8.9 ± 2.2	10213 ± 2592	0.19 ± 0.01	2.1 ± 0.9	51.3 ± 5.2	0.69 ± 0.41

Supplementary Figure 9 | Dye analysis, Alexa Fluor 647

DNA origami imaged using Abberior Star 635p with Trolox

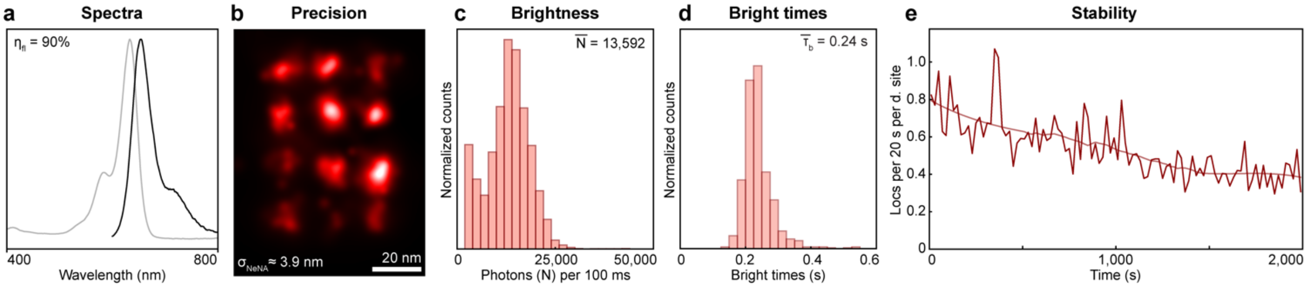

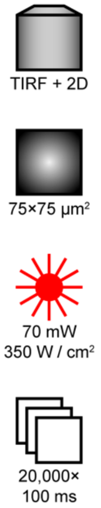

f DNA origami average results for all buffer conditions

Dye	Ex. max. (nm)	Em. max. (nm)	QY	Ext. (×10³ M⁻¹ cm⁻¹)	Buffer additive	Precision (nm)	Photons per 100 ms	Bright time (s)	Signal to background ratio	Localization drop (%)	Destroyed binding sites (%)
Abberior Star 635p	638	651	0.9	120	-	4.9 ± 0.5	11526 ± 825	0.24 ± 0.02	2.1 ± 0.1	45.6 ± 20.3	14.0 ± 6.9
					Trolox	4.5 ± 0.7	12731 ± 1037	0.23 ± 0.01	2.3 ± 0.3	53.3 ± 24.8	17.9 ± 11.6
					PCA PCD Trolox	7.0 ± 0.3	3813 ± 227	0.40 ± 0.00	0.9 ± 0.0	81.6 ± 2.3	68.6 ± 4.3

U2OS Nup96 imaged using Abberior Star 635p with Trolox

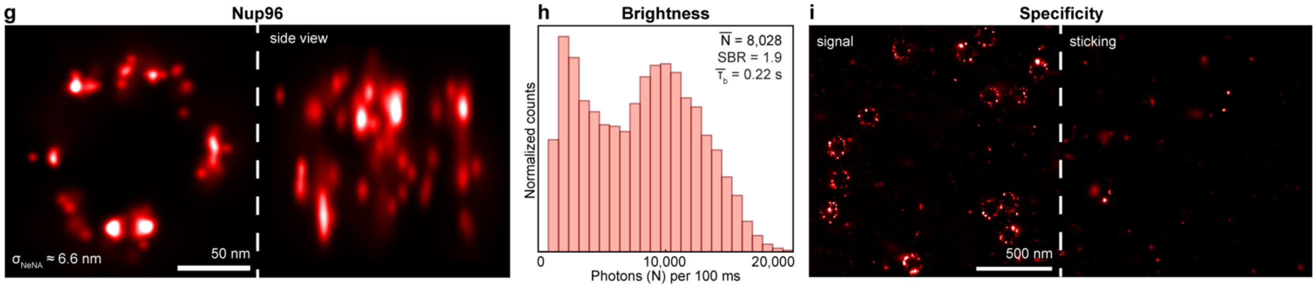

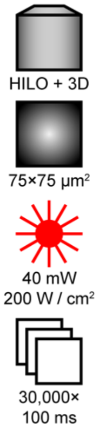

j U2OS Nup96 average results with Trolox

Dye	Buffer additive	Precision (nm)	Photons per 100 ms	Bright time (s)	Signal to background ratio	Localization drop (%)	Relative specificity
Abberior Star 635p	Trolox	8.0 ± 1.3	9609 ± 2651	0.22 ± 0.01	1.7 ± 0.3	6.7 ± 6.1	0.37 ± 0.29

Supplementary Figure 10 | Dye analysis, Abberior Star 635p

DNA origami imaged using Atto643 with Trolox

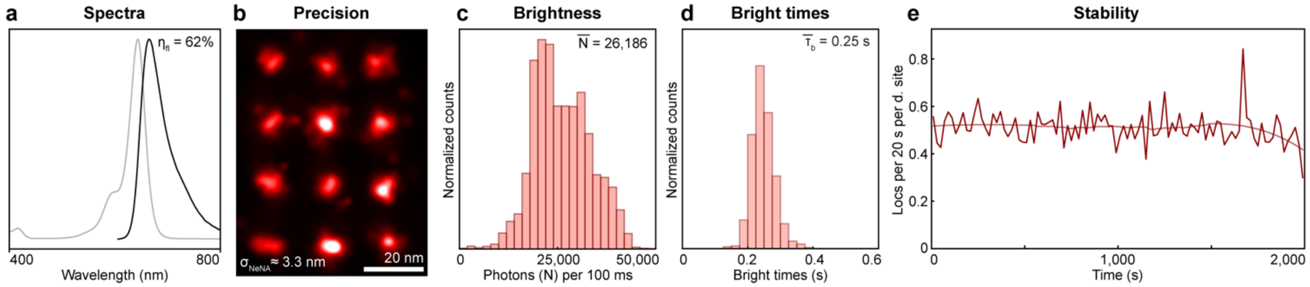

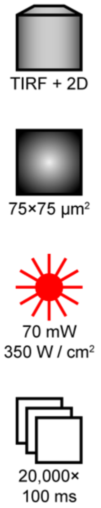

f DNA origami average results for all buffer conditions

Dye	Ex. max. (nm)	Em. max. (nm)	QY	Ext. (×10³ M⁻¹ cm⁻¹)	Buffer additive	Precision (nm)	Photons per 100 ms	Bright time (s)	Signal to background ratio	Localization drop (%)	Destroyed binding sites (%)
Atto643	643	665	0.62	150	-	3.9 ± 0.7	18148 ± 2632	0.25 ± 0.03	3.3 ± 0.8	11.4 ± 10.3	1.0 ± 0.9
					Trolox	3.4 ± 0.3	23327 ± 6241	0.25 ± 0.01	4.3 ± 0.7	12.1 ± 4.5	1.9 ± 0.5
					PCA PCD Trolox	4.7 ± 1.1	15525 ± 8480	0.24 ± 0.02	3.1 ± 1.3	5.0 CI (0.0, 10.3)	0.9 CI (0.0, 1.8)

U2OS Nup96 imaged using Atto643 with Trolox

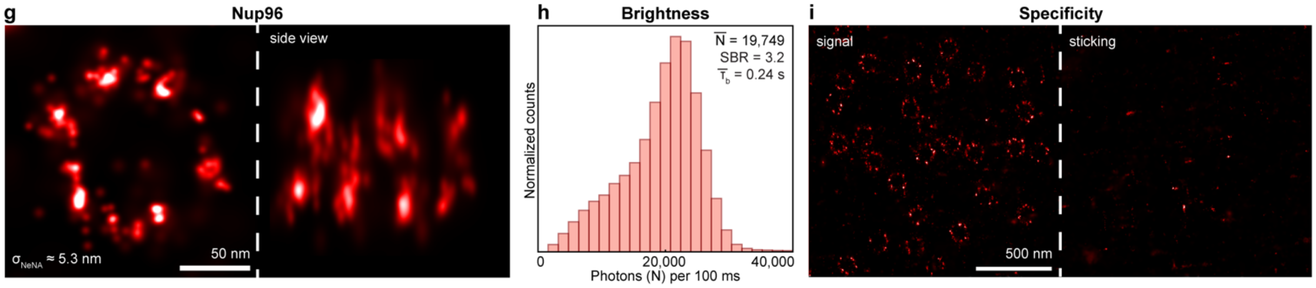

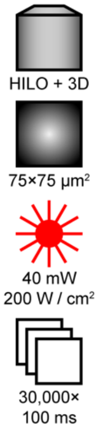

j U2OS Nup96 average results with Trolox

Dye	Buffer additive	Precision (nm)	Photons per 100 ms	Bright time (s)	Signal to background ratio	Localization drop (%)	Relative specificity
Atto643	Trolox	5.3 ± 1.3	14803 ± 4342	0.26 ± 0.02	2.8 ± 0.5	5.8 CI (0.0, 13.9)	0.66 ± 0.13

Supplementary Figure 11 | Dye analysis, Atto643

DNA origami imaged using Atto647N with Trolox

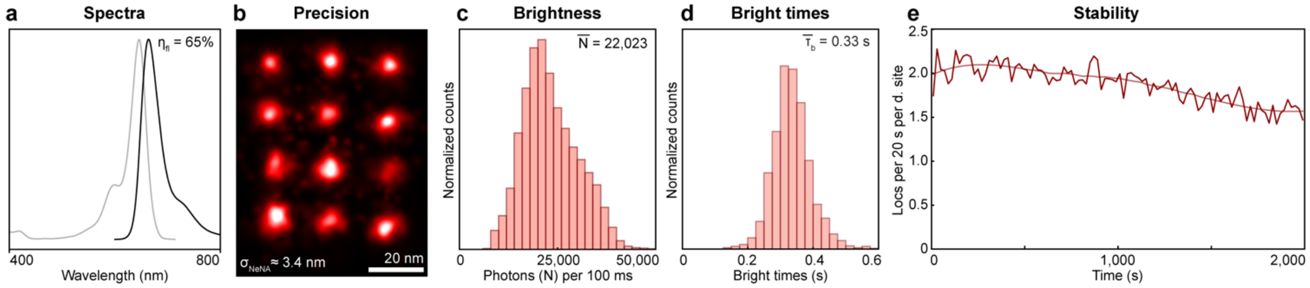

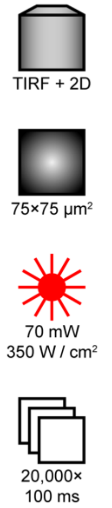

f DNA origami average results for all buffer conditions

Dye	Ex. max. (nm)	Em. max. (nm)	QY	Ext. (×10³ M⁻¹ cm⁻¹)	Buffer additive	Precision (nm)	Photons per 100 ms	Bright time (s)	Signal to background ratio	Localization drop (%)	Destroyed binding sites (%)
Atto647N	646	664	0.65	150	-	4.6 ± 0.4	13071 ± 2270	0.21 ± 0.01	2.9 ± 0.1	2.0 CI (0.0, 5.5)	7.0 ± 4.0
					Trolox	3.4 ± 0.1	18448 ± 3523	0.32 ± 0.01	4.0 ± 0.1	18.7 ± 9.2	4.0 ± 1.9
					PCA PCD Trolox	4.5 ± 0.5	14699 ± 5124	0.29 ± 0.04	2.9 ± 0.6	1.6 CI (0.0, 4.4)	3.2 ± 1.1

U2OS Nup96 imaged using Atto647N with Trolox

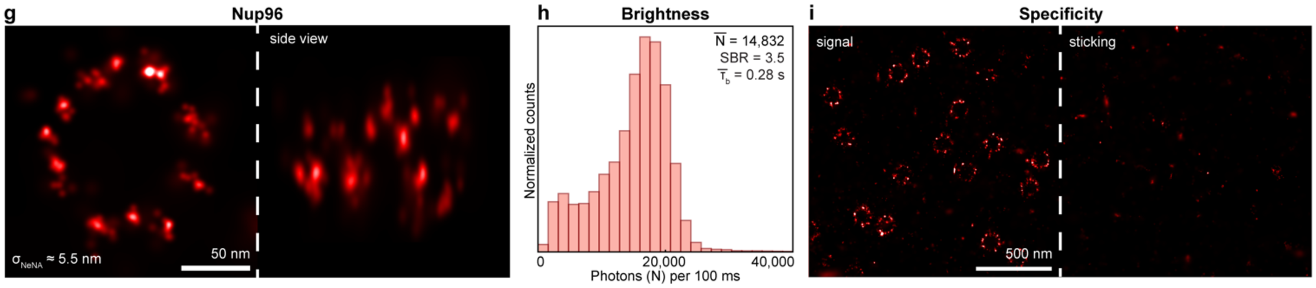

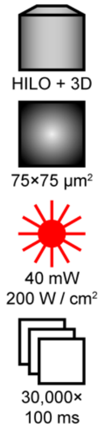

j U2OS Nup96 average results with Trolox

Dye	Buffer additive	Precision (nm)	Photons per 100 ms	Bright time (s)	Signal to background ratio	Localization drop (%)	Relative specificity
Atto647N	Trolox	6.1 ± 0.8	15396 ± 1254	0.27 ± 0.01	3.2 ± 0.3	4.8 ± 2.7	0.38 ± 0.03

Supplementary Figure 12 | Dye analysis, Atto647N

DNA origami imaged using Atto655

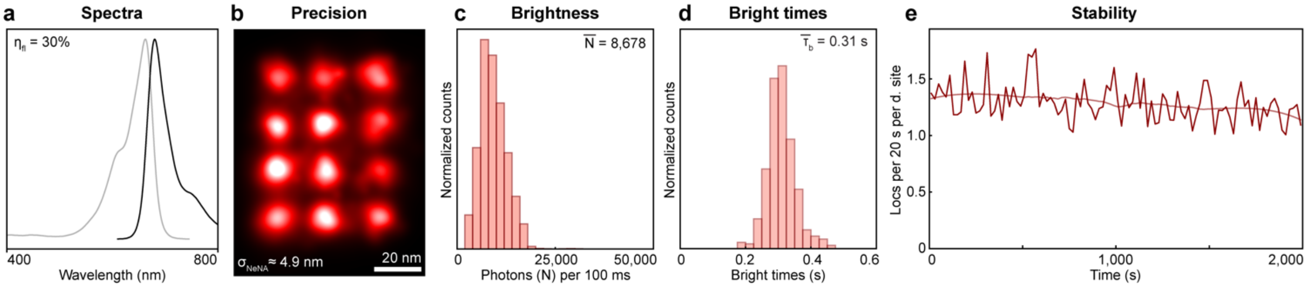

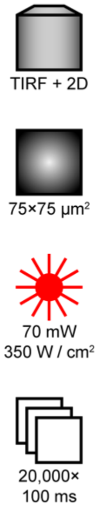

f DNA origami average results for all buffer conditions

Dye	Ex. max. (nm)	Em. max. (nm)	QY	Ext. (×10³ M⁻¹ cm⁻¹)	Buffer additive	Precision (nm)	Photons per 100 ms	Bright time (s)	Signal to background ratio	Localization drop (%)	Destroyed binding sites (%)
Atto655	663	680	0.3	125	-	5.8 ± 1.7	8273 ± 2732	0.27 ± 0.04	1.7 ± 0.1	8.9 ± 4.0	4.0 ± 2.7
					Trolox	13.2 ± 7.0	3047 ± 2388	0.24 ± 0.02	0.8 ± 0.4	7.2 CI (0.0, 14.6)	4.8 ± 3.8
					PCA PCD Trolox	16.6 ± 6.7	1147 ± 107	0.19 ± 0.04	0.4 ± 0.0	12.3 CI (0.0, 29.7)	4.8 ± 3.4

U2OS Nup96 imaged using Atto655

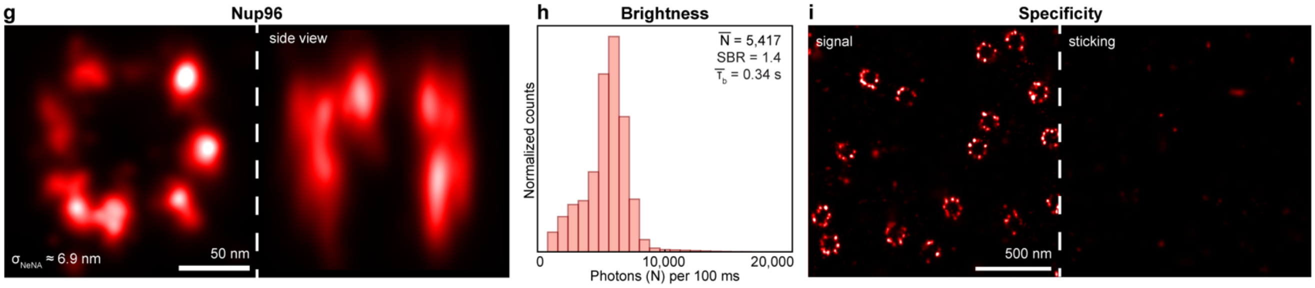

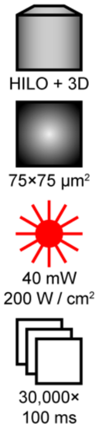

j U2OS Nup96 average results

Dye	Buffer additive	Precision (nm)	Photons per 100 ms	Bright time (s)	Signal to background ratio	Localization drop (%)	Relative specificity
Atto655	-	7.3 ± 0.5	5763 ± 1736	0.31 ± 0.04	1.2 ± 0.1	2.3 CI (0.0, 6.3)	1.44 ± 0.42

Supplementary Figure 13 | Dye analysis, Atto655

DNA origami imaged using CF640R

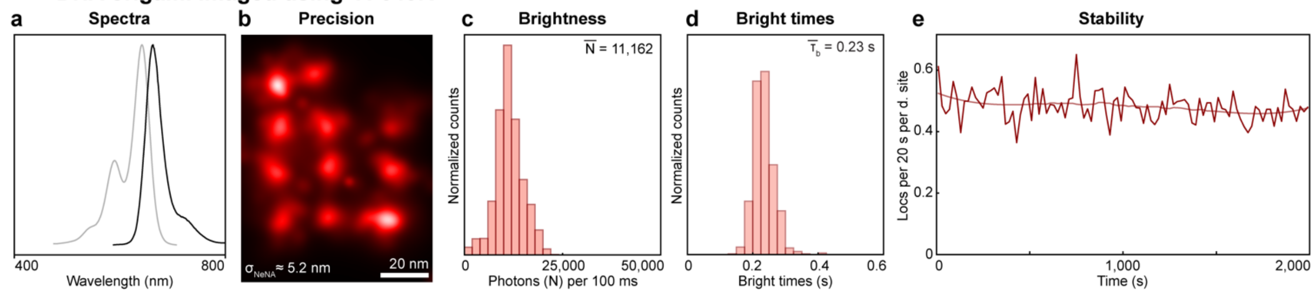

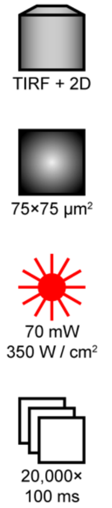

f DNA origami average results for all buffer conditions

Dye	Ex. max. (nm)	Em. max. (nm)	QY	Ext. (×10³ M⁻¹ cm⁻¹)	Buffer additive	Precision (nm)	Photons per 100 ms	Bright time (s)	Signal to background ratio	Localization drop (%)	Destroyed binding sites (%)
CF640R	642	662	-	105	-	5.5 ± 1.0	12024 ± 3240	0.22 ± 0.01	2.2 ± 0.2	7.5 ± 2.5	2.7 ± 1.2
					Trolox	6.8 ± 0.9	7951 ± 1883	0.22 ± 0.01	1.8 ± 0.1	21.8 ± 0.8	3.3 ± 1.3
					PCA PCD Trolox	10.4 ± 0.1	4176 ± 858	0.18 ± 0.01	1.1 ± 0.4	9.9 ± 0.5	3.7 ± 1.1

U2OS Nup96 imaged using CF640R

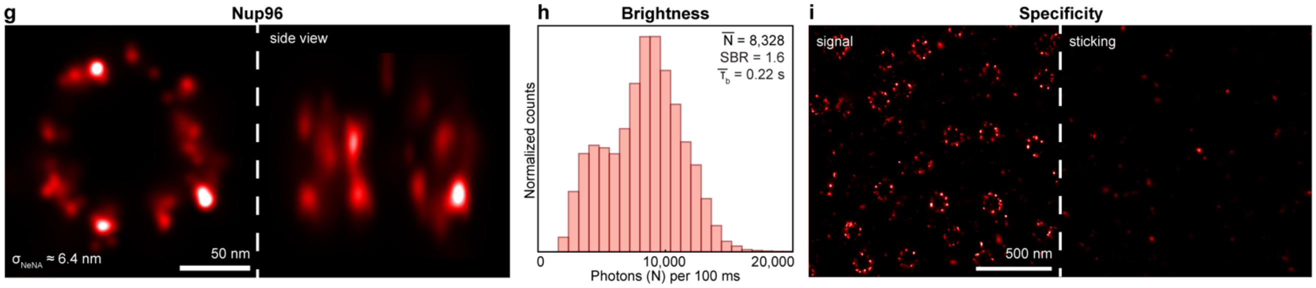

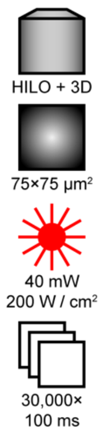

j U2OS Nup96 average results

Dye	Buffer additive	Precision (nm)	Photons per 100 ms	Bright time (s)	Signal to background ratio	Localization drop (%)	Relative specificity
CF640R	-	7.3 ± 0.8	7539 ± 2114	0.21 ± 0.01	1.3 ± 0.3	5.7 ± 5.0	1.15 ± 0.39

Supplementary Figure 14 | Dye analysis, CF640R

DNA origami imaged using CF660R

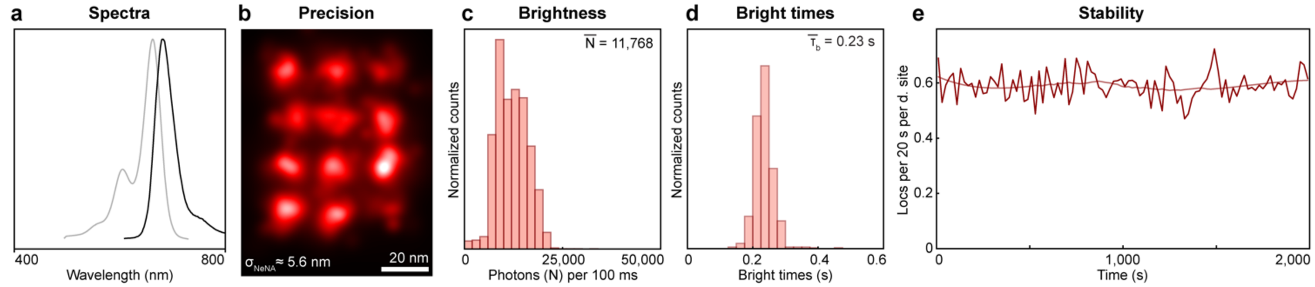

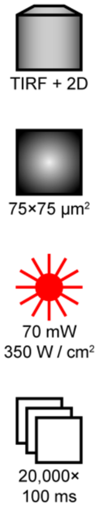

f DNA origami average results for all buffer conditions

Dye	Ex. max. (nm)	Em. max. (nm)	QY	Ext. (×10^3 M^{-1} cm^{-1})	Buffer additive	Precision (nm)	Photons per 100 ms	Bright time (s)	Signal to background ratio	Localization drop (%)	Destroyed binding sites (%)
CF660R	663	682	-	100	-	6.6 ± 0.9	10399 ± 1309	0.21 ± 0.02	2.3 ± 0.3	4.2 ± 3.8	2.8 ± 0.8
					Trolox	6.7 ± 0.8	8894 ± 1493	0.21 ± 0.02	2.2 ± 0.0	11.3 ± 2.5	1.8 ± 0.5
					PCA PCD Trolox	9.4 ± 1.3	6200 ± 468	0.18 ± 0.01	1.6 ± 0.5	7.8 ± 6.2	2.8 ± 1.3

U2OS Nup96 imaged using CF660R

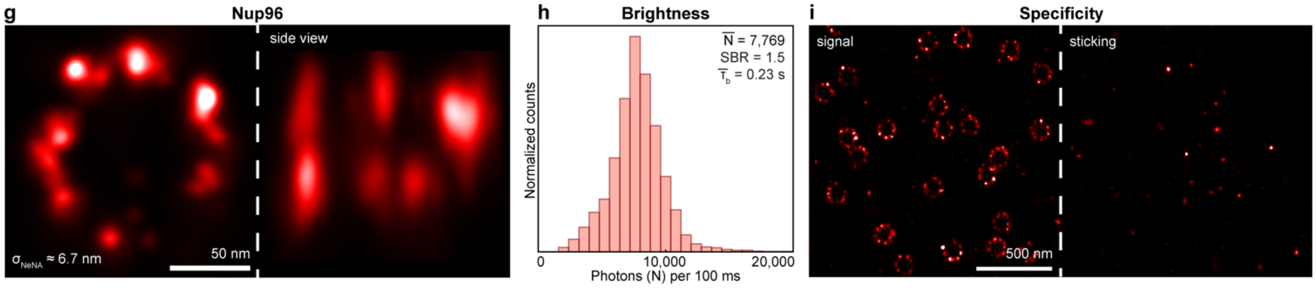

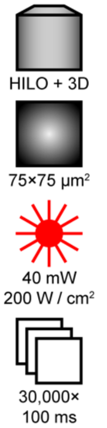

j U2OS Nup96 average results

Dye	Buffer additive	Precision (nm)	Photons per 100 ms	Bright time (s)	Signal to background ratio	Localization drop (%)	Relative specificity
CF660R	-	6.8 ± 0.1	7219 ± 583	0.24 ± 0.01	1.6 ± 0.1	1.8 ± 1.7	0.83 ± 0.20

Supplementary Figure 15 | Dye analysis, CF660R

DNA origami imaged using Cy5 with PCA / PCD / Trolox

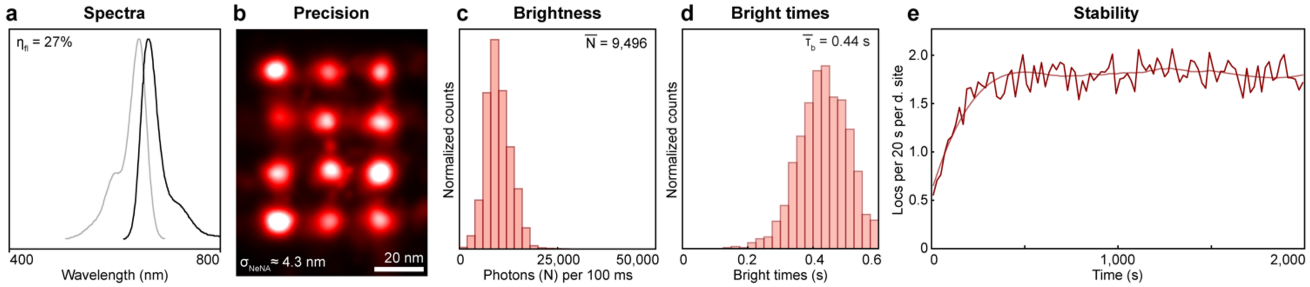

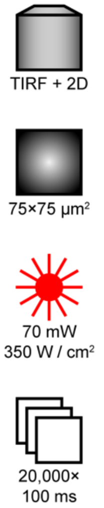

f **DNA origami average results for all buffer conditions**

Dye	Ex. max. (nm)	Em. max. (nm)	QY	Ext. (×10³ M⁻¹ cm⁻¹)	Buffer additive	Precision (nm)	Photons per 100 ms	Bright time (s)	Signal to background ratio	Localization drop (%)	Destroyed binding sites (%)
Cy5	651	670	0.27	250	-	12.5 ± 4.1	3268 ± 524	0.19 ± 0.02	1.2 ± 0.1	41.9 ± 14.1	8.7 ± 4.4
					Trolox	9.2 ± 0.4	5118 ± 2106	0.19 ± 0.01	1.5 ± 0.2	35.7 ± 4.2	8.8 ± 3.0
					PCA PCD Trolox	4.7 ± 0.6	7801 ± 1722	0.42 ± 0.02	2.0 ± 0.1	0.0 ± 0.0	2.1 ± 1.8

U2OS Nup96 imaged using Cy5 with PCA / PCD / Trolox

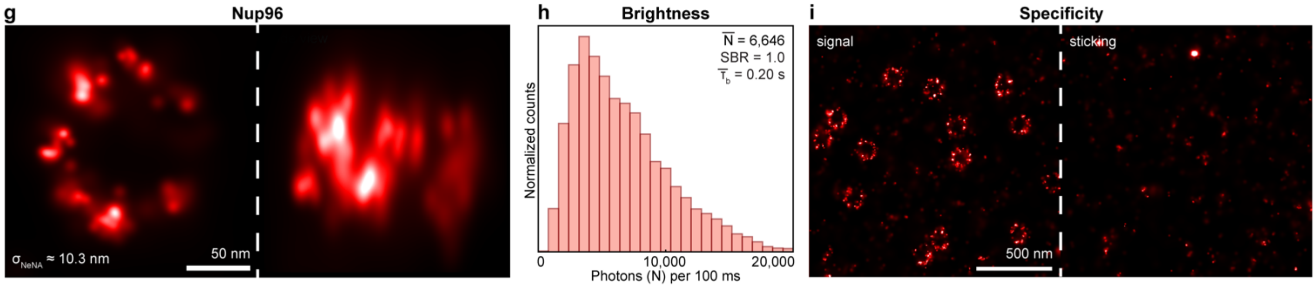

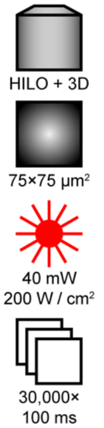

j **U2OS Nup96 average results with PCA / PCD / Trolox**

Dye	Buffer additive	Precision (nm)	Photons per 100 ms	Bright time (s)	Signal to background ratio	Localization drop (%)	Relative specificity
Cy5	PCA PCD Trolox	10.0 ± 2.0	6031 ± 540	0.21 ± 0.03	1.1 ± 0.2	54.4 ± 18.0	0.72 ± 0.14

Supplementary Figure 16 | Dye analysis, Cy5

DNA origami imaged using Cy5B with PCA / PCD / Trolox

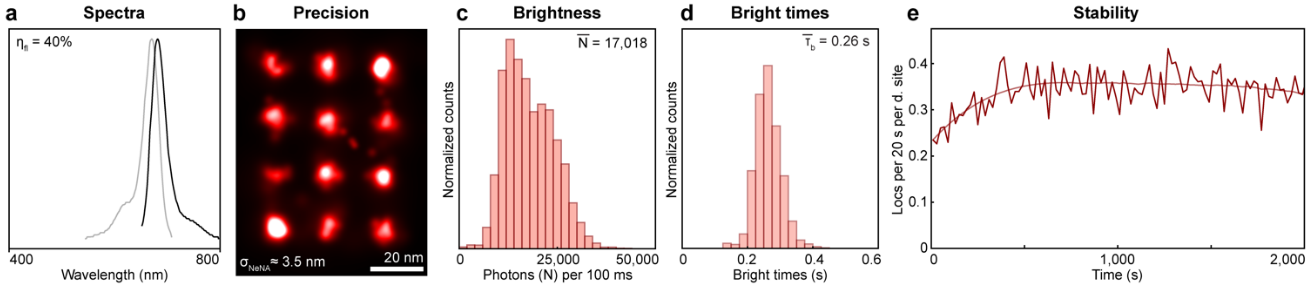

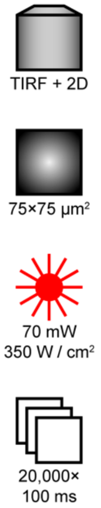

f DNA origami average results for all buffer conditions

Dye	Ex. max. (nm)	Em. max. (nm)	QY	Ext. (×10³ M⁻¹ cm⁻¹)	Buffer additive	Precision (nm)	Photons per 100 ms	Bright time (s)	Signal to background ratio	Localization drop (%)	Destroyed binding sites (%)
Cy5B	671	682	0.4	241	-	4.9 ± 0.1	16123 ± 2066	0.19 ± 0.01	3.1 ± 0.7	17.1 ± 0.6	3.3 ± 0.6
					Trolox	4.1 ± 0.4	20804 ± 4255	0.21 ± 0.02	3.7 ± 0.9	11.5 ± 6.9	2.8 ± 0.5
					PCA PCD Trolox	4.0 ± 0.4	17090 ± 2765	0.25 ± 0.02	2.9 ± 0.5	0.3 CI (0.0,0.8)	2.1 ± 0.5

U2OS Nup96 imaged using Cy5B with PCA / PCD / Trolox

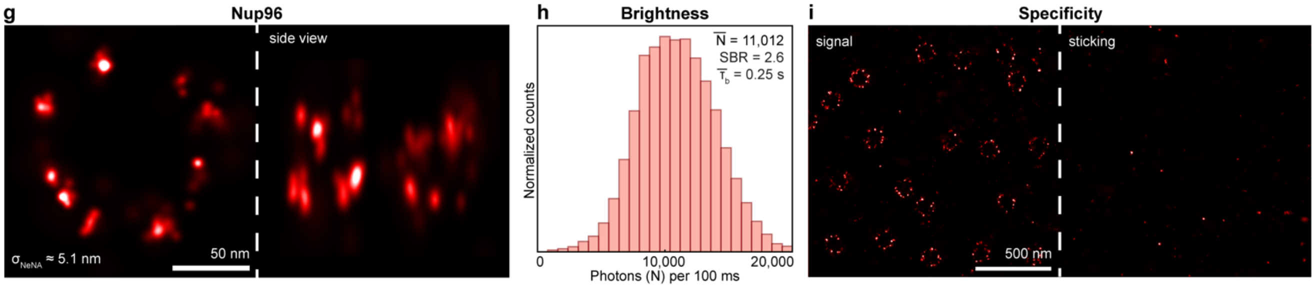

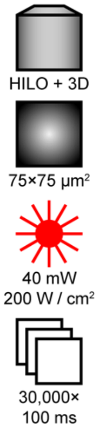

j U2OS Nup96 average results with PCA / PCD / Trolox

Dye	Buffer additive	Precision (nm)	Photons per 100 ms	Bright time (s)	Signal to background ratio	Localization drop (%)	Relative specificity
Cy5B	PCA PCD Trolox	5.6 ± 0.5	9483 ± 2068	0.25 ± 0.01	2.2 ± 0.6	20.9 ± 18.4	0.77 ± 0.16

Supplementary Figure 17 | Dye analysis, Cy5B

DNA origami imaged using Janelia Fluor 646

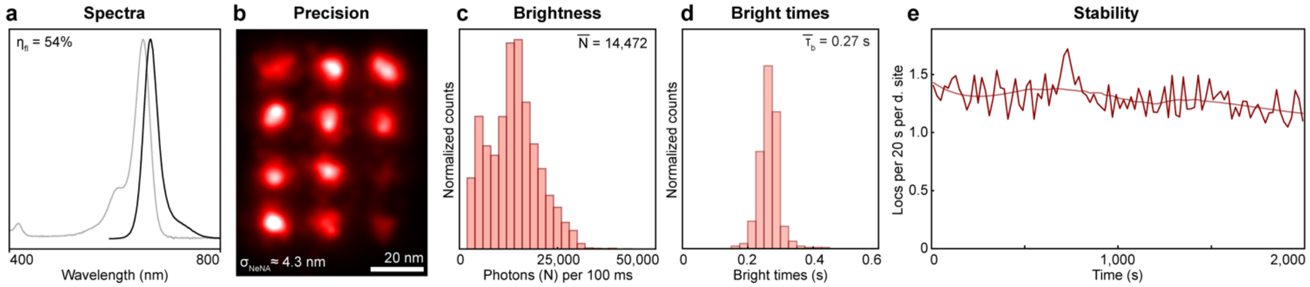

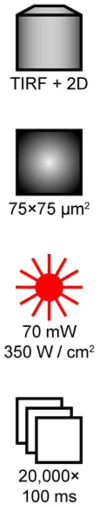

f DNA origami average results for all buffer conditions

Dye	Ex. max. (nm)	Em. max. (nm)	QY	Ext. (×10^3 M^{-1} cm^{-1})	Buffer additive	Precision (nm)	Photons per 100 ms	Bright time (s)	Signal to background ratio	Localization drop (%)	Destroyed binding sites (%)
Janelia Fluor 646	646	664	0.54	152	-	4.3 ± 0.1	14440 ± 3596	0.25 ± 0.03	2.6 ± 0.4	23.3 ± 9.1	5.6 ± 2.6
					Trolox	5.8 ± 2.0	12660 ± 4664	0.25 ± 0.04	2.6 ± 0.4	14.4 CI (0.0, 29.0)	2.5 CI (0.0, 6.0)
					PCA PCD Trolox	5.6 ± 0.2	10743 ± 1597	0.25 ± 0.01	2.4 ± 0.7	1.6 CI (0.0, 3.9)	4.0 ± 0.1

U2OS Nup96 imaged using Janelia Fluor 646

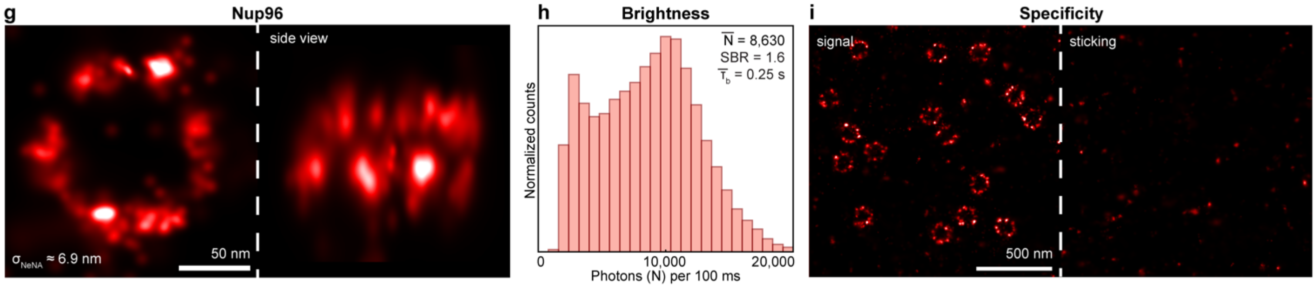

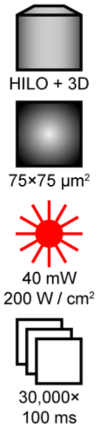

j U2OS Nup96 average results

Dye	Buffer additive	Precision (nm)	Photons per 100 ms	Bright time (s)	Signal to background ratio	Localization drop (%)	Relative specificity
Janelia Fluor 646	-	9.3 ± 3.0	8221 ± 1152	0.26 ± 0.01	1.7 ± 0.1	7.0 ± 6.8	0.43 ± 0.06

Supplementary Figure 18 | Dye analysis, Janelia Fluor 646

Supplementary Table 1 | Imaging parameters

Target & imaging round	Laser [nm]	Laser power at objective [mW]	Laser power density [W/cm^2]	Imager	Imager conc. [pM]	Buffer and additive	Number of frames	Imaging time [min]
**DNA origami, barcode & reference**	560	35	175	R1-Cy3B<br>R3-Cy3B	500<br>500	B with PCA, PCD, Trolox	10,000	16.7
**DNA origami, blue dyes**	488	20	100	R1-Dye of interest	500	B,<br>all conditions	20,000	33.3
**DNA origami, green dyes**	560	35	175	R1-Dye of interest	500	B,<br>all conditions	20,000	33.3
**DNA origami, red dyes**	640	70	350	R1-Dye of interest	300 (Cy5, Atto643, Atto647N),<br>500 (rest)	B,<br>all conditions	20,000	33.3
**DNA origami, var. powers, CF488**	488	10, 20, 40, 60, 80, 100	50, 100, 200, 300, 400, 500	R1-CF488A	500	B with Trolox	20,000	33.3
**DNA origami, var. powers, Cy3B**	560	35, 50, 100, 150	175, 250, 500, 750	R1-Cy3B	500	B with PCA, PCD, Trolox	20,000	33.3
**DNA origami, var. powers, Atto643**	640	35, 70, 100, 150, 200	175, 350, 500, 750, 1000	R1-Atto643	500	B with Trolox	20,000	33.3
**U2OS NPC, reference**	560	20	100	R1-Cy3B	80	C with PCA, PCD, Trolox	30,000	50
**U2OS NPC, blue dyes**	488	15	75	R1-Dye of interest	80 - 120	C with optimal additive	30,000	50
**U2OS NPC, green dyes**	560	20	100	R1-Dye of interest	80 - 100	C with optimal additive	30,000	50
**U2OS NPC, red dyes**	640	40	200	R1-Dye of interest	40 - 80 (Atto643),<br>20 - 50 (Atto647N),<br>80 -100 (rest)	C with optimal additive	30,000	50
**U2OS Tom20, reference**	560	20	100	R1-Cy3B	100	C with PCA, PCD, Trolox	30,000	50
**U2OS Tom20, blue dyes**	488	15	75	R1-Dye of interest	100	C with optimal additive	30,000	50
**U2OS Tom20, green dyes**	560	20	100	R1-Dye of interest	100	C with optimal additive	30,000	50
**U2OS Tom20, red dyes**	640	40	200	R1-Dye of interest	100	C with optimal additive	30,000	50
**Neurons, round 1**	488<br>560<br>640	15<br>20<br>40	75<br>100<br>200	R1-CF488A<br>R4-Cy3B<br>R6-Atto643	200<br>150<br>350	C with Trolox	30,000	50
**Neurons, round 2**	488<br>560<br>640	15<br>20<br>40	75<br>100<br>200	R5-CF488A<br>R3-Cy3B<br>R2-Atto643	150<br>300<br>100	C with Trolox	30,000	50

Imaging conditions for all measurements. The exposure time was set to 100 ms for all measurements. DNA origami "dye of interest" experiments were carried out with only buffer B, buffer B with Trolox, and buffer B with PCA, PCD, and Trolox for all dyes. U2OS NPC and Tom20 "dye of interest" experiments were carried out with the optimal buffer additive for each dye, specified in **Supplementary Figures 1-18**.

Supplementary Table 2 | Sequences

Imager name	Imager Sequence	Docking strand sequence
R1	AGGAGGA	TCCTCCTCCTCCTCCTCCT
R2	TGGTGGT	ACCACCACCACCACCACCA
R3	GAGAGAG	CTCTCTCTCTCTCTCTCTC
R4	TGTGTGT	ACACACACACACACACACA
R5	GAAGAAG	CTTCTTCTTCTTCTTCTTC
R6	TTGTTGTT	AACAACAACAACAACAACAA

Supplementary Table 3 | Antibodies

Antibody	Vendor	Information	Dilution
Mouse monoclonal (SAP7F407) anti-Bassoon	Enzo	Cat# ADI-VAM-PS003-F; RRID:AB_11181058	1 in 200
Rabbit polyclonal anti-VGAT	invitrogen	Cat# PA5-27569; RRID:AB_2545045	1 in 300
Rabbit monoclonal (EPR15581-54) anti-Tom20	Abcam	Cat# ab186735; RRID:AB_2889972	1 in 200
Mouse monoclonal (69H10) anti-Neurofilament L	Synaptic Systems	Cat# 171011; RRID:AB_2891275	1 in 200
Mouse monoclonal (42/B) anti-βII Spectrin	BD Biosciences	Cat# 612562; RRID:AB_399853	1 in 100
sdAb anti-PSD95 (1B2)	NanoTag Biotechnologies	Cat# N3705	1 in 200
sdAb anti-GFP (1H1)	NanoTag Biotechnologies	Cat# N0305	1 in 200
sdAb anti-Mouse IgG (10A4)	NanoTag Biotechnologies	Cat# N2005	1 in 300
sdAb anti-Rabbit IgG (10E10)	NanoTag Biotechnologies	Cat# N2405	1 in 300
Multiplexing Blocker Mouse	NanoTag Biotechnologies	Cat# K0102-50	1 in 200
Multiplexing Blocker Mouse	NanoTag Biotechnologies	Cat# K0202-50	1 in 200

Supplementary Table 4 | Cross-talk in 3-channel imaging

Dye added	Atto643	Cy3b	CF488
Number of locs detected in red channel	1145912	894	1720
Number of locs detected in green channel	1497	1798626	6253
Number of locs detected in blue channel	4818	14587	993884
Total number of locs	1152227	1814107	1001857
Percent of locs detected in red channel	99.45	0.05	0.17
Percent of locs detected in green channel	0.13	99.15	0.62
Percent of locs detected in blue channel	0.42	0.80	99.20
Ratio red / correct	1.000000	0.000497	0.001731
Ratio green / correct	0.001306	1.000000	0.006291
Ratio blue / correct	0.004205	0.008110	1.000000

Supplementary Table 5 | DNA origami scaffold sequence

TTCCCTTCCTTTCTCGCCACGTTCGCCGGCTTTCCCCGTCAAGCTCTAAATCGGGGGCTCCCTTTAGGGTTCCGA
TTTAGTGCTTTACGGCACCTCGACCCCAAAAAACTTGATTTGGGTGATGGTTCACGTAGTGGGCCATCGCCCTG
ATAGACGGTTTTTCGCCCTTTGACGTTGGAGTCCACGTTCTTTAATAGTGGACTCTTGTTCCAAACTGGAACAA
CACTCAACCCTATCTCGGGCTATTCTTTTGATTTATAAGGGATTTTGCCGATTTCGGAACCACCATCAAACAGGAT
TTTCGCCTGCTGGGGCAAACCAGCGTGGACCGCTTGCTGCAACTCTCTCAGGGCCAGGCGGTGAAGGGCAAT
CAGCTGTTGCCCGTCTCACTGGTGAAAAGAAAAACCACCCTGGCGCCCAATACGCAAACCGCCTCTCCCCGCG
CGTTGGCCGATTCATTAATGCAGCTGGCACGACAGGTTTCCCGACTGGAAAGCGGGCAGTGAGCGCAACGCA
ATTAATGTGAGTTAGCTCACTCATTAGGCACCCCAGGCTTTACACTTTATGCTTCCGGCTCGTATGTTGTGTGGA
ATTGTGAGCGGATAACAATTTCACACAGGAAACAGCTATGACCATGATTACGAATTCGAGCTCGGTACCCGGGG
ATCCTCTAGAGTCGACCTGCAGGCATGCAAGCTTGGCACTGGCCGTCGTTTTACAACGTCGTGACTGGGAAAA
CCCTGGCGTTACCCAACTTAATCGCCTTGCAGCACATCCCCCTTTCGCCAGCTGGCGTAATAGCGAAGAGGCCC
GCACCGATCGCCCTTCCCAACAGTTGCGCAGCCTGAATGGCGAATGGCGCTTTGCCTGGTTTCCGGCACCAGA
AGCGGTGCCGGAAAGCTGGCTGGAGTGCGATCTTCCTGAGGCCGATACTGTCGTCGTCCCCTCAAACTGGCAG
ATGCACGGTTACGATGCGCCCATCTACACCAACGTGACCTATCCCATTACGGTCAATCCGCCGTTTGTTCCCACG
GAGAATCCGACGGGTTGTTACTCGCTCACATTTAATGTTGATGAAAGCTGGCTACAGGAAGGCCAGACGCGAA
TTATTTTTGATGGCGTTCCTATTGGTTAAAAAATGAGCTGATTTAACAAAAATTTAATGCGAATTTTAACAAAATA
TTAACGTTTACAATTTAAATATTTGCTTATACAATCTTCCTGTTTTTGGGGCTTTTCTGATTATCAACCGGGGTACA
TATGATTGACATGCTAGTTTTACGATTACCGTTCATCGATTCTCTTGTTTGCTCCAGACTCTCAGGCAATGACCTG
ATAGCCTTTGTAGATCTCTCAAAAATAGCTACCCTCTCCGGCATTAATTTATCAGCTAGAACGGTTGAATATCATAT
TGATGGTGATTTGACTGTCTCCGGCCTTTCTCACCCTTTTGAATCTTTACCTACACATTACTCAGGCATTGCATTTA
AAATATATGAGGGTTCTAAAAATTTTTATCCTTGCGTTGAAATAAAGGCTTCTCCCGCAAAAGTATTACAGGGTC
ATAATGTTTTTGGTACAACCGATTTAGCTTTATGCTCTGAGGCTTTATTGCTTAATTTTGCTAATTCTTTGCCTTGC
CTGTATGATTTATTGGATGTTAATGCTACTACTATTAGTAGAATTGATGCCACCTTTTCAGCTCGCGCCCCAAATGA
AAATATAGCTAAACAGGTTATTGACCATTTGCGAAATGTATCTAATGGTCAAACTAAATCTACTCGTTCGCAGAAT
TGGGAATCAACTGTTATATGGAATGAAACTTCCAGACACCGTACTTTAGTTGCATATTTAAAACATGTTGAGCTA
CAGCATTATATTCAGCAATTAAGCTCTAAGCCATCCGCAAAAATGACCTCTTATCAAAAGGAGCAATTAAAGGTA
CTCTCTAATCCTGACCTGTTGGAGTTTGCTTCCGGTCTGGTTCGCTTTGAAGCTCGAATTAAAACGCGATATTTG
AAGTCTTTCGGGCTTCCTCTTAATCTTTTTGATGCAATCCGCTTTGCTTCTGACTATAATAGTCAGGGTAAAGACC
TGATTTTTGATTTATGGTCATTCTCGTTTTCTGAACTGTTTAAAGCATTTGAGGGGGATTCAATGAATATTTATGA
CGATTCCGCAGTATTGGACGCTATCCAGTCTAAACATTTTACTATTACCCCCTCTGGCAAAACTTCTTTTGCAAAA
GCCTCTCGCTATTTTGGTTTTTATCGTCGTCTGGTAAACGAGGGTTATGATAGTGTTGCTCTTACTATGCCTCGTA
ATTCCTTTTGGCGTTATGTATCTGCATTAGTTGAATGTGGTATTCCTAAATCTCAACTGATGAATCTTTCTACCTGT
AATAATGTTGTTCCGTTAGTTCGTTTTATTAACGTAGATTTTTCTTCCCAACGTCCTGACTGGTATAATGAGCCAG
TTCTTAAAATCGCATAAGGTAATTCACAATGATTAAAGTTGAAATTAAACCATCTCAAGCCCAATTTACTACTCGT
TCTGGTGTTTCTCGTCAGGGCAAGCCTTATTCACTGAATGAGCAGCTTTGTTACGTTGATTTGGGTAATGAATAT
CCGGTTCTTGTCAAGATTACTCTTGATGAAGGTCAGCCAGCCTATGCGCCTGGTCTGTACACCGTTCATCTGTCC

TCTTTCAAAGTTGGTCAGTTCGGTTCCCTTATGATTGACCGTCTGCGCCTCGTTCCGGCTAAGTAACATGGAGCA
GGTCGCGGATTTCGACACAATTTATCAGGCGATGATACAAATCTCCGTTGTACTTTGTTTCGCGCTTGGTATAATC
GCTGGGGGTCAAAGATGAGTGTTTTAGTGTATTCTTTTGCCTCTTTCGTTTTAGGTTGGTGCCTTCGTAGTGGCA
TTACGTATTTTACCCGTTTAATGGAAACTTCCTCATGAAAAAGTCTTTAGTCCTCAAAGCCTCTGTAGCCGTTGCT
ACCCTCGTTCCGATGCTGTCTTTCGCTGCTGAGGGTGACGATCCCGCAAAAGCGGCCTTTAACTCCCTGCAAGC
CTCAGCGACCGAATATATCGGTTATGCGTGGGCGATGGTTGTTGTCATTGTCGGCGCAACTATCGGTATCAAGCT
GTTTAAGAAATTCACCTCGAAAGCAAGCTGATAAACCGATACAATTAAAGGCTCCTTTTGGAGCCTTTTTTTTG
GAGATTTTCAACGTGAAAAAATTATTATTCGCAATTCCTTTAGTTGTTCCTTTCTATTCTCACTCCGCTGAAACTG
TTGAAAGTTGTTTAGCAAAATCCCATACAGAAAATTCATTTACTAACGTCTGGAAAGACGACAAAACTTTAGATC
GTTACGCTAACTATGAGGGCTGTCTGTGGAATGCTACAGGCGTTGTAGTTTGTACTGGTGACGAAACTCAGTGT
TACGGTACATGGGTTCCTATTGGGCTTGCTATCCCTGAAAATGAGGGTGGTGGCTCTGAGGGTGGCGGTTCTG
AGGGTGGCGGTTCTGAGGGTGGCGGTACTAAACCTCCTGAGTACGGTGATACACCTATTCCGGGCTATACTTAT
ATCAACCCTCTCGACGGCACTTATCCGCCTGGTACTGAGCAAAACCCCGCTAATCCTAATCCTTCTCTTGAGGAG
TCTCAGCCTCTTAATACTTTCATGTTTCAGAATAATAGGTTCCGAAATAGGCAGGGGGCATTAACTGTTTATACG
GGCACTGTTACTCAAGGCACTGACCCCGTTAAAACTTATTACCAGTACACTCCTGTATCATCAAAAGCCATGTAT
GACGCTTACTGGAACGGTAAATTCAGAGACTGCGCTTTCCATTCTGGCTTTAATGAGGATTTATTTGTTTGTGAA
TATCAAGGCCAATCGTCTGACCTGCCTCAACCTCCTGTCAATGCTGGCGGCGGCTCTGGTGGTGGTTCTGGTGG
CGGCTCTGAGGGTGGTGGCTCTGAGGGTGGCGGTTCTGAGGGTGGCGGCTCTGAGGGAGGCGGTTCCGGTG
GTGGCTCTGGTTCCGGTGATTTTGATTATGAAAAGATGGCAAACGCTAATAAGGGGGCTATGACCGAAAATGCC
GATGAAAACGCGCTACAGTCTGACGCTAAAGGCAAACTTGATTCTGTCGCTACTGATTACGGTGCTGCTATCGA
TGGTTTCATTGGTGACGTTTCCGGCCTTGCTAATGGTAATGGTGCTACTGGTGATTTTGCTGGCTCTAATTCCCA
AATGGCTCAAGTCGGTGACGGTGATAATTCACCTTTAATGAATAATTTCCGTCAATATTTACCTTCCCTCCCTCAAT
CGGTTGAATGTCGCCCTTTTGTCTTTGGCGCTGGTAAACCATATGAATTTTCTATTGATTGTGACAAAATAAACTT
ATTCCGTGGTGTCTTTGCGTTTCTTTTATATGTTGCCACCTTTATGTATGTATTTTCTACGTTTGCTAACATACTGCG
TAATAAGGAGTCTTAATCATGCCAGTTCTTTTGGGTATTCCGTTATTATTGCGTTTCCTCGGTTTCCTTCTGGTAAC
TTTGTTCGGCTATCTGCTTACTTTTCTTAAAAAGGGCTTCGGTAAGATAGCTATTGCTATTTCATTGTTTCTTGCTC
TTATTATTGGGCTTAACTCAATTCTTGTGGGTTATCTCTCTGATATTAGCGCTCAATTACCCTCTGACTTTGTTCAG
GGTGTTCAGTTAATTCTCCCGTCTAATGCGCTTCCCTGTTTTTATGTTATTCTCTCTGTAAAGGCTGCTATTTTCAT
TTTTGACGTTAAACAAAAAATCGTTTCTTATTTGGATTGGGATAAATAATATGGCTGTTTATTTTGTAACTGGCAA
ATTAGGCTCTGGAAAGACGCTCGTTAGCGTTGGTAAGATTCAGGATAAAATTGTAGCTGGGTGCAAAATAGCA
ACTAATCTTGATTTAAGGCTTCAAAACCTCCCGCAAGTCGGGAGGTTCGCTAAAACGCCTCGCGTTCTTAGAAT
ACCGGATAAGCCTTCTATATCTGATTTGCTTGCTATTGGGCGCGGTAATGATTCCTACGATGAAAATAAAAACGG
CTTGCTTGTTCTCGATGAGTGCGGTACTTGGTTTAATACCCGTTCTTGGAATGATAAGGAAAGACAGCCGATTAT
TGATTGGTTTCTACATGCTCGTAAATTAGGATGGGATATTATTTTTCTTGTTCAGGACTTATCTATTGTTGATAAAC
AGGCGCGTTCTGCATTAGCTGAACATGTTGTTTATTGTCGTCGTCTGGACAGAATTACTTTACCTTTTGTCGGTA
CTTTATATTCTCTTATTACTGGCTCGAAAATGCCTCTGCCTAAATTACATGTTGGCGTTGTTAAATATGGCGATTCT
CAATTAAGCCCTACTGTTGAGCGTTGGCTTTATACTGGTAAGAATTTGTATAACGCATATGATACTAAACAGGCTT
TTTCTAGTAATTATGATTCCGGTGTTTATTCTTATTTAACGCCTTATTTATCACACGGTCGGTATTTCAAACCATTAA
ATTTAGGTCAGAAGATGAAATTAACTAAAATATATTTGAAAAAGTTTTCTCGCGTTCTTTGTCTTGCGATTGGATT
TGCATCAGCATTTACATATAGTTATATAACCCAACCTAAGCCGGAGGTTAAAAAGGTAGTCTCTCAGACCTATGAT
TTTGATAAATTCACTATTGACTCTTCTCAGCGTCTTAATCTAAGCTATCGCTATGTTTTCAAGGATTCTAAGGGAA
AATTAATTAATAGCGACGATTTACAGAAGCAAGGTTATTCACTCACATATATTGATTTATGTACTGTTTCCATTAAA
AAAGGTAATTCAAATGAAATTGTTAAATGTAATTAATTTTGTTTTCTTGATGTTTGTTTCATCATCTTCTTTTGCTC
AGGTAATTGAAATGAATAATTCGCCTCTGCGCGATTTTGTAACTTGGTATTCAAAGCAATCAGGCGAATCCGTTA
TTGTTTCTCCCGATGTAAAAGGTACTGTTACTGTATATTCATCTGACGTTAAACCTGAAAATCTACGCAATTTCTTT
ATTTCTGTTTTACGTGCAAATAATTTTGATATGGTAGGTTCTAACCCTTCCATTATTCAGAAGTATAATCCAAACAA
TCAGGATTATATTGATGAATTGCCATCATCTGATAATCAGGAATATGATGATAATTCCGCTCCTTCTGGTGGTTTCT
TTGTTCCGCAAAATGATAATGTTACTCAAACTTTTAAAATTAATAACGTTCGGGCAAAGGATTTAATACGAGTTG
TCGAATTGTTTGTAAAGTCTAATACTTCTAAATCCTCAAATGTATTATCTATTGACGGCTCTAATCTATTAGTTGTTA
GTGCTCCTAAAGATATTTTAGATAACCTTCCTCAATTCCTTTCAACTGTTGATTTGCCAACTGACCAGATATTGATT
GAGGGTTTGATATTTGAGGTTCAGCAAGGTGATGCTTTAGATTTTTCATTTGCTGCTGGCTCTCAGCGTGGCAC
TGTTGCAGGCGGTGTTAATACTGACCGCCTCACCTCTGTTTTATCTTCTGCTGGTGGTTCGTTCGGTATTTTTAAT
GGCGATGTTTTAGGGCTATCAGTTCGCGCATTAAAGACTAATAGCCATTCAAAAATATTGTCTGTGCCACGTATTC
TTACGCTTTCAGGTCAGAAGGGTTCTATCTCTGTTGGCCAGAATGTCCCTTTTATTACTGGTCGTGTGACTGGTG
AATCTGCCAATGTAAATAATCCATTTCAGACGATTGAGCGTCAAAATGTAGGTATTTCCATGAGCGTTTTTCCTGT
TGCAATGGCTGGCGGTAATATTGTTCTGGATATTACCAGCAAGGCCGATAGTTTG
