# Supplementary material for: The DNA-PAINT palette: a comprehensive performance analysis of fluorescent dyes
Source: Nat Methods. 2024 Aug 7;21(9):1755–62. doi: 10.1038/s41592-024-02374-8 (PMC11399092; doi:10.1038/s41592-024-02374-8)
Supplement: Supplementary file 2 — Reporting Summary [file 41592_2024_2374_MOESM2_ESM.pdf]

nature portfolio

Corresponding author(s): Ralf Jungmann

Last updated by author(s): May 29, 2024

Reporting Summary

Nature Portfolio wishes to improve the reproducibility of the work that we publish. This form provides structure for consistency and transparency in reporting. For further information on Nature Portfolio policies, see our Editorial Policies and the Editorial Policy Checklist.

Statistics

For all statistical analyses, confirm that the following items are present in the figure legend, table legend, main text, or Methods section.

n/a	Confirmed	
☐	☒	The exact sample size (*n*) for each experimental group/condition, given as a discrete number and unit of measurement
☐	☒	A statement on whether measurements were taken from distinct samples or whether the same sample was measured repeatedly
☒	☐	The statistical test(s) used AND whether they are one- or two-sided<br>*Only common tests should be described solely by name; describe more complex techniques in the Methods section.*
☒	☐	A description of all covariates tested
☒	☐	A description of any assumptions or corrections, such as tests of normality and adjustment for multiple comparisons
☐	☒	A full description of the statistical parameters including central tendency (e.g. means) or other basic estimates (e.g. regression coefficient) AND variation (e.g. standard deviation) or associated estimates of uncertainty (e.g. confidence intervals)
☒	☐	For null hypothesis testing, the test statistic (e.g. *F*, *t*, *r*) with confidence intervals, effect sizes, degrees of freedom and *P* value noted<br>*Give P values as exact values whenever suitable.*
☒	☐	For Bayesian analysis, information on the choice of priors and Markov chain Monte Carlo settings
☒	☐	For hierarchical and complex designs, identification of the appropriate level for tests and full reporting of outcomes
☒	☐	Estimates of effect sizes (e.g. Cohen's *d*, Pearson's *r*), indicating how they were calculated

Our web collection on statistics for biologists contains articles on many of the points above.

Software and code

Policy information about availability of computer code

Data collection	Raw microscopy data was acquired using µManager version 2.0.1 (Edelstein, A., Amodaj, N., Hoover, K., Vale, R. & Stuurman, N. Curr. Protoc. Mol. Biol. 14.20 (2010)).
Data analysis	All data were analyzed using the open source software Picasso versions 0.6.0 - 0.6.9 (Schnitzbauer, J., Strauss, M. T., Schlichthaerle, T., Schueder, F., & Jungmann, R. (2017). Super-resolution microscopy with DNA-PAINT. Nature Protocols, 12(6), 1198–1228. http://doi.org/10.1038/nprot.2017.024),<br>and custom code (10.5281/zenodo.10960858 or https://github.com/PhilippSteen/DNA-PAINT_analysis and https://github.com/PhilippSteen/Affine-transformation)

For manuscripts utilizing custom algorithms or software that are central to the research but not yet described in published literature, software must be made available to editors and reviewers. We strongly encourage code deposition in a community repository (e.g. GitHub). See the Nature Portfolio guidelines for submitting code & software for further information.

Data

Policy information about availability of data

All manuscripts must include a data availability statement. This statement should provide the following information, where applicable:

- Accession codes, unique identifiers, or web links for publicly available datasets
- A description of any restrictions on data availability
- For clinical datasets or third party data, please ensure that the statement adheres to our policy

Localization data from this study are available at 10.5281/zenodo.10960858. Raw microscopy data obtained during this study are available from the corresponding author on reasonable request.

Human research participants

Policy information about studies involving human research participants and Sex and Gender in Research.

Reporting on sex and gender	n/a
Population characteristics	n/a
Recruitment	n/a
Ethics oversight	n/a

Note that full information on the approval of the study protocol must also be provided in the manuscript.

Field-specific reporting

Please select the one below that is the best fit for your research. If you are not sure, read the appropriate sections before making your selection.

☒ Life sciences ☐ Behavioural & social sciences ☐ Ecological, evolutionary & environmental sciences

For a reference copy of the document with all sections, see nature.com/documents/nr-reporting-summary-flat.pdf

Life sciences study design

All studies must disclose on these points even when the disclosure is negative.

Sample size	Sample size n is defined as the number of independent microscopy experiments performed.
Data exclusions	No data were excluded.
Replication	All replications (3 independent experiments per parameter investigated) were successful.
Randomization	n/a, no grouping of experiments or samples was performed.
Blinding	n/a, no grouping of experiments or samples was performed.

Reporting for specific materials, systems and methods

We require information from authors about some types of materials, experimental systems and methods used in many studies. Here, indicate whether each material, system or method listed is relevant to your study. If you are not sure if a list item applies to your research, read the appropriate section before selecting a response.

Materials & experimental systems

n/a	Involved in the study
☐	☒ Antibodies
☐	☒ Eukaryotic cell lines
☒	☐ Palaeontology and archaeology
☐	☒ Animals and other organisms
☒	☐ Clinical data
☒	☐ Dual use research of concern

Methods

n/a	Involved in the study
☒	☐ ChIP-seq
☒	☐ Flow cytometry
☒	☐ MRI-based neuroimaging

Antibodies

Antibodies used

1) Mouse monoclonal (SAP7F407) anti-Bassoon, Enzo (Cat# ADI-VAM-PS003-F; RRID:AB_11181058), dilution 1 in 200
2) Rabbit polyclonal anti-VGAT, Invitrogen (Cat# PA5-27569; RRID:AB_2545045), dilution 1 in 300
3) Rabbit monoclonal (EPR15581-54) anti-Tom20, Abcam (Cat# ab186735; RRID:AB_2889972), dilution 1 in 200
4) Mouse monoclonal (69H10) anti-Neurofilament L, Synaptic Systems (Cat# 171011; RRID:AB_2891275), dilution 1 in 200
5) Mouse monoclonal (42/B) anti-βII Spectrin, BD Biosciences (Cat# 612562; RRID:AB_399853), dilution 1 in 100
6) sdAb anti-PSD95 (1B2), NanoTag Biotechnologies (Cat# N3705), dilution 1 in 200
7) sdAb anti-GFP (1H1), NanoTag Biotechnologies (Cat# N0305), dilution 1 in 200
8) sdAb anti-Mouse IgG (10A4), NanoTag Biotechnologies (Cat# N2005), dilution 1 in 300
9) sdAb anti-Rabbit IgG (10E10), NanoTag Biotechnologies (Cat# N2405), dilution 1 in 300
10) Multiplexing Blocker Mouse, NanoTag Biotechnologies (Cat# K0102-50), dilution 1 in 200
11) Multiplexing Blocker Mouse, NanoTag Biotechnologies (Cat# K0202-50), dilution 1 in 200

Validation

Antibodies and Nanobodies were verified by Manufacturers by Immunofluorescence and Western blot to ensure that the binders bind to the antigen stated.

Eukaryotic cell lines

Policy information about cell lines and Sex and Gender in Research

Cell line source(s)

U-2 OS-CRISPR-Nup96-mEGFP cells were obtained from the Ellenberg and Ries lab (Reference: https://doi.org/10.1038/s41592-019-0574-9).

Authentication

The cell lines were not authenticated.

Mycoplasma contamination

All cell lines have been tested negative for mycoplasma contamination.

Commonly misidentified lines
(See ICLAC register)

No commonly misidentified cell lines were used.

Animals and other research organisms

Policy information about studies involving animals; ARRIVE guidelines recommended for reporting animal research, and Sex and Gender in Research

Laboratory animals

Wild-type Wistar rat pregnant mothers and pups (Rattus norvegicus), P2.

Wild animals

No wild animals were used in this study.

Reporting on sex

Animals of both sexes were used in this study.

Field-collected samples

No field-collected samples were used in this study.

Ethics oversight

Animal experiments were approved by the local authority, the Lower Saxony State Office for Consumer Protection and Food Safety (Niedersächsisches Landesamt für Verbraucherschutz und Lebensmittelsicherheit).

Note that full information on the approval of the study protocol must also be provided in the manuscript.
